# Genetic sequence-based prediction of long-range chromatin interactions suggests a potential role of short tandem repeat sequences in genome organization

**DOI:** 10.1186/s12859-017-1624-x

**Published:** 2017-04-18

**Authors:** Sarvesh Nikumbh, Nico Pfeifer

**Affiliations:** 1 Computational Biology & Applied Algorithmics, Max Planck Institute for Informatics, Saarland Informatics Campus, Building E1.4, Saarbruecken, D-66123 Germany; 2 Present address: Department of Computer Science, University of Tübingen, Sand 14, Tübingen, D-72076 Germany

**Keywords:** Long-range interactions prediction, Support vector machines, Multitask learning, Hi-C, Visualizations

## Abstract

**Background:**

Knowing the three-dimensional (3D) structure of the chromatin is important for obtaining a complete picture of the regulatory landscape. Changes in the 3D structure have been implicated in diseases. While there exist approaches that attempt to predict the long-range chromatin interactions, they focus only on interactions between specific genomic regions — the promoters and enhancers, neglecting other possibilities, for instance, the so-called structural interactions involving intervening chromatin.

**Results:**

We present a method that can be trained on 5C data using the genetic sequence of the candidate loci to predict potential genome-wide interaction partners of a particular locus of interest. We have built locus-specific support vector machine (SVM)-based predictors using the oligomer distance histograms (ODH) representation. The method shows good performance with a mean test AUC (area under the receiver operating characteristic (ROC) curve) of 0.7 or higher for various regions across cell lines GM12878, K562 and HeLa-S3. In cases where any locus did not have sufficient candidate interaction partners for model training, we employed multitask learning to share knowledge between models of different loci. In this scenario, across the three cell lines, the method attained an average performance increase of 0.09 in the AUC. Performance evaluation of the models trained on 5C data regarding prediction on an independent high-resolution Hi-C dataset (which is a rather hard problem) shows 0.56 AUC, on average. Additionally, we have developed new, intuitive visualization methods that enable interpretation of sequence signals that contributed towards prediction of locus-specific interaction partners. The analysis of these sequence signals suggests a potential general role of short tandem repeat sequences in genome organization.

**Conclusions:**

We demonstrated how our approach can 1) provide insights into sequence features of locus-specific interaction partners, and 2) also identify their cell-line specificity. That our models deem short tandem repeat sequences as discriminative for prediction of potential interaction partners, suggests that they could play a larger role in genome organization. Thus, our approach can (a) be beneficial to broadly understand, at the sequence-level, chromatin interactions and higher-order structures like (meta-) topologically associating domains (TADs); (b) study regions omitted from existing prediction approaches using various information sources (e.g., epigenetic information); and (c) improve methods that predict the 3D structure of the chromatin.

**Electronic supplementary material:**

The online version of this article (doi:10.1186/s12859-017-1624-x) contains supplementary material, which is available to authorized users.

## Background

It is well known that chromatin, a complex of DNA and proteins, is packed in three-dimensional (3D) space inside the nucleus of the cell in a highly regulated fashion. The spatial conformation of chromosomes is governed by certain principles [1–3]. The structure of chromatin depends on the functional state of the cell (viz. normal/diseased) and gene activity among other cellular properties. Thus, a better understanding of 3D chromatin structure and the underlying mechanisms determining this structure helps in gaining an enhanced comprehension of many genomic functions. With the advent of chromosome conformation capture (3C)-based technologies in the last decade, starting with 3C itself in 2002, chromosome conformation capture-on-chip and circular chromosome conformation capture (both abbreviated as 4C), and 3C-carbon copy (5C) in 2006, chromatin interaction analysis by paired-end tag sequencing (ChIA-PET), 2009 [4–8], more recently Hi-C [9] and in situ high-resolution Hi-C [10] which is still quite expensive, genome-wide analysis of the interaction profiles is now possible [11]. Studies have revealed a correlation between long-range chromatin interactions and the functional state of the cell, e.g., in [12] and more generally, cell-type specificity as evidenced by [11]. These long-range interactions comprise pairs of loci that are close in space, but not necessarily close in sequence. The spatial co-localization of different chromosomal regions (*cis* as well as *trans*) can be due to a mix of factors, for example specific, direct contacts between two loci, nonspecific binding as a result of the packing of the chromatin fibre or co-localization due to functional association or having the same subnuclear structure [13].

Any long-range interaction (i.e., interaction between genomic loci separated by >1 or 2 mega base pairs) can typically occur to bring about or increase the likelihood of a certain activity at either of these loci itself (e.g., between an enhancer and a promoter region) or so that they can trigger or play an important role in any activity (e.g., facilitating binding of a protein) taking place at these loci or in their neighborhood on the genome. Knowledge of which loci interact over a long-range and evaluating the effect of such interactions can help us further our understanding of genome regulation and organization. Thus, it is of general interest to be able to predict whether a given pair of loci lying very far apart on the chromosome would interact. There exist machine learning-based approaches for predicting such long-range interactions between enhancer and promoter regions, for example, [14]. They combine the contact information output by a chromatin interaction experiment with various information sources, for example, epigenetic information [14], to make these predictions, but these approaches leave out genomic regions for which such information is not available. A sequence-level model, in addition to primarily furthering our understanding of chromatin interactions at the most basic level, can also be useful to study any genomic region including the ones omitted by other approaches. Having a model that can predict, based on sequence information alone, whether two regions are likely to interact has several potential applications. One is to use the predicted label as additional information for the prediction of boundaries of topologically associating domains (TADs) [15]. Another is to assist methods that predict the 3D structure of the chromosome from Hi-C data [16].

As a word of caution, since the genetic sequence is only the primary level at which genomic function and organization information is encoded, it is apparent that higher levels of modifications will have the final say towards these chromatin interactions, more so for cell line-specificity. In other words, one would not expect a model using sequence information alone to outshine one that (also) utilizes additional information sources in terms of prediction accuracy. But, a sequence-level model has its advantages as already stated. Thus, we would like to stress upon our aim in performing this study:

- (a) Answer the question: To what extent can the genetic sequence alone predict these long-range chromosomal interactions? We report on various computational experiments, using our genetic-sequence based prediction method, to establish that the DNA sequence is informative to identify potential interaction partners of a given genomic locus, and
- (b) Understand the characteristic sequence features underlying such long-range interactions. This is achieved with the help of our two new visualization methods that aid in interpreting the sequence signals that contributed towards predicting locus-specific interaction partners and reveal interesting biological connections.

In general, we believe that such an approach using sequence-level information could be useful to study sequence peculiarities among the interaction partners of a particular locus. Our approach could augment existing methods for prediction of 3D chromatin structure and also TAD boundary predictions methods.

### Approach

In this study we built a method based on support vector machines (SVMs) [17] to predict which genomic loci potentially interact with a given locus based on the genetic sequence. In a nutshell, we do the following: given a contact matrix delineating interactions between various genomic loci, we build a predictor for a locus of interest (LoI) from the contact matrix. This predictor learns the characteristics of the genomic loci that happen to significantly interact with the LoI as against the set of loci that do not. Thus, we build a predictor per locus. Such locus-specific predictors that use the genetic sequence information at these loci have the potential to uncover peculiarities of the interacting partners of this particular locus which can be useful to understand interactions at the sequence level. Such an understanding can guide us in our efforts to know the role-players at the genetic level and comprehend mechanisms of higher levels of chromatin organization viz. TADs and their hierarchies, and compartments. When dealing with contact matrices output from a chromatin interaction experiment where a large population of non-synchronized cells are studied, such an approach can still give us a holistic view.

We analyzed 5C contact matrices for three *human* cell lines — GM12878, K562 and HeLa-S3 — and demonstrated that the genetic sequence is predictive of the long-range interactions. Additionally, we utilized these locus-specific models, that were trained on the 5C data, to independently predict potential interaction partners across the chromosome for the same LoI. This computational validation is done on high-resolution Hi-C datasets from Rao et al. [10]. Our new visualization methods help to intuitively visualize the sequence features that proved useful for discerning the interaction partners of a LoI from those that do not interact with it, consequently rendering our models to be more than black boxes. Due to the models being locus-specific, one is also able to compare the sequence features found useful by a model (using our visualization) for a locus in one cell line to those found useful by the model for the same locus in another cell line. This is discussed in “Identifying cell-line specific characteristic signals among (non-)interactors of the same locus in different cell lines” section.

## Results and discussion

**Fig. 1:**
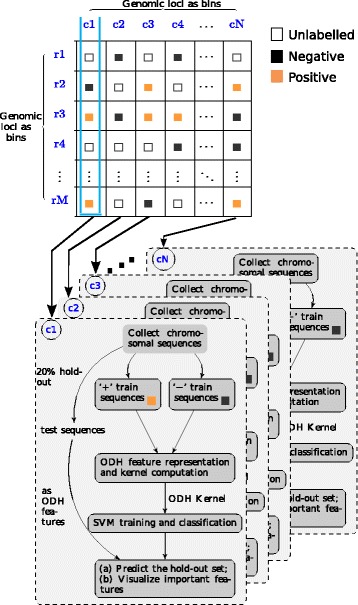
Pipeline for predicting locus-specific long-range chromatin interactions using the genetic sequence. In the contact matrix, cells denoted by filled *orange boxes* correspond to loci that are called significantly interacting with the LoI in all replicates of any experiment profiling chromatin interactions. This constitutes the positive set of sequences for the corresponding classifier. Those denoted by filled *black boxes* correspond to loci that are not called significantly interacting in any of the replicates. This constitutes the negative set of sequences for the corresponding classifier. This leaves those loci which are called significantly interacting in at least one, but not in all of the replicates. They are visualized by *unfilled boxes* and are not used by the classifier. The genomic loci along the columns of the contact matrix (c1, c2, c3,...,cN) are the LoI for which we build locus-specific classifiers

Applicable to information on long-range contacts facilitated by a 4C, 5C or a Hi-C experiment, we describe our pipeline and the corresponding computational experiments performed on data from a 5C experiment [18] that detects interactions between a group of transcription start site (TSS)-containing regions (TCRs [18]) and distal enhancers in the three cell lines GM12878, K562 and HeLa-S3. Here, for each cell line, we built a separate classifier per TCR. Given the set of loci, for which the contact frequency with the TCR of interest (ToI) is known (from the contact matrix), we trained an SVM [17] which, when presented with a new, unseen locus, can classify it as positive or negative (i.e., interacting with the ToI or not). We use string kernels, which provide a measure of similarity between sequences, in conjunction with the SVM. The aim was to build a pipeline with the best possible locus-specific classifiers (a separate classifier for each LoI/ToI), and also be able to determine subsequently, which sequence features were most important for any classifier to distinguish between the positive and the negative set of genomic loci corresponding to the LoI. Our pipeline is shown in Fig. 1 and described in the “Methodical details” section.

### Prediction of long-range chromatin interactions is possible from the sequence alone using non-linear SVMs

To evaluate the potential of the DNA sequence to serve as the sole information source in predicting the long-range interactions, we selected ten regions per cell line. For each cell line, these are the top 10 regions when ranked based on the number of positive examples available for them (see Supplementary Table S1 in Additional file 1). In each model, the varied-length sequences were represented as fixed-length feature vectors using the oligomer distance histograms (ODH) [19] representation. This represents any sequence by the histograms of distances between *K*-mers in the sequence (see ‘Methodical details” section for more details). We performed experiments with *K*-mer values 3 and 5 and the maximum distance between *K*-mers as 100. Intuitively, *K*-mer value 5 encodes more specificity towards the set of sequences in a collection for a model while *K*-mer value 3 maintains relative generality. Once these are fixed, the ODH kernel has no other hyper-parameters to be tuned.

Table 1 summarily shows good test AUC (area under the ROC curve) values for all studied regions in all the three cell lines resulting from our 5-fold nested cross validation. Furthermore, our pipeline is also capable of handling imbalances in the data. For all the regions in our computational experiments, the positive class is in minority. We report performances with data imbalance handled (see “Methodical details”).

**Table 1:** Locus information for regions and prediction performances

*R*	TCR	#TP	#NP	Test AUC				*R*	TCR	#TP	#NP	Test AUC			
				A	B	C	D					A	B	C	D
GM12878															
0	chr7:115847372-115857098	63	226	0.7417	0.7538	0.8979	0.9042	5	chr7:90224881-90229046	34	122	0.8078	0.8307	0.9221	0.9118
1	chr7:115890993-115892266	56	234	0.7141	0.7341	0.8876	0.8960	6	chr7:116434729-116454408	33	292	0.7785	0.7787	0.7308	0.7036
2	chr7:115861595-115870968	52	252	0.7346	0.7763	0.9152	0.9376	7	chr7:90337078-90341001	32	158	0.8163	0.8275	0.9286	0.9324
3	chr5:131722317-131724751	39	91	0.6122	0.6547	0.8666	0.8286	8	chr22:32162110-32166713	31	127	0.7779	0.7832	0.7789	0.7738
4	chr5:131892428-131895867	34	80	0.5971	0.6343	0.8889	0.8543	9	chr21:34819525-34821921	30	201	0.6704	0.6694	0.7157	0.6901
K562															
0	chr22:32764253-32784733	46	105	0.8163	0.8121	0.9308	0.9382	5	chr7:89787744-89795672	35	118	0.8546	0.8648	0.8566	0.8727
1	chr22:32920308-32927723	45	109	0.6808	0.7242	0.7744	0.7972	6	chrX:153625659-153635385	34	46	0.8501	0.8495	0.8044	0.8184
2	chr22:32012966-32043914	42	104	0.7145	0.7324	0.8378	0.8599	7	chr22:32170492-32188129	32	97	0.7456	0.7146	0.8003	0.8228
3	chr21:35242603-35256847	39	150	0.7321	0.725	0.7251	0.7407	8	chr22:32740683-32750950	32	112	0.7167	0.7582	0.8836	0.9166
4	chr7:115847372-115857098	37	238	0.7521	0.7756	0.7765	0.7908	9	chr11:5721056-5732713	31	85	0.671	0.76	0.7345	0.7545
HeLa-S3															
0	chr7:115847372-115857098	98	207	0.6914	0.7111	0.8007	0.8228	5	chr7:115861595-115870968	40	284	0.6624	0.732	0.8964	0.9114
1	chr7:116434729-116454408	71	211	0.73	0.7674	0.8573	0.8738	6	chr22:32170492-32188129	40	102	0.677	0.755	0.8245	0.8590
2	chr22:32920308-32927723	53	109	0.644	0.6369	0.7338	0.7091	7	chr22:32053085-32061138	37	115	0.6018	0.6420	0.7886	0.7991
3	chr7:115890993-115892266	50	243	0.6817	0.7225	0.907	0.9162	8	chr22:33262063-33266567	37	112	0.5634	0.6564	0.8449	0.8491
4	chr7:89787744-89795672	49	108	0.8108	0.8007	0.8005	0.8084	9	chr21:34750664-34761738	37	147	0.7194	0.7294	0.7053	0.7273

#TruePeaks (#TP) and #NonPeaks (#NP) for all the studied genomic regions (column ‘R’) for the three cell lines (GM12878, K562 and HeLa-S3). Columns marked ‘A’, ‘B’, ‘C’ and ‘D’ show the mean test AUC values with oligomer length 3 and 5 respectively for two settings: Individual tasks (‘A’ and ‘B’) and Multiple tasks (‘C’ and ‘D’). Refer “Pipeline for predicting long-range chromatin interactions”, “Prediction of long-range chromatin interactions is possible from the sequence alone using non-linear SVMs” and “Multitask learning (MTL) helps mitigate issue of having too few interacting partners per locus” sections for more information

**Fig. 2:**
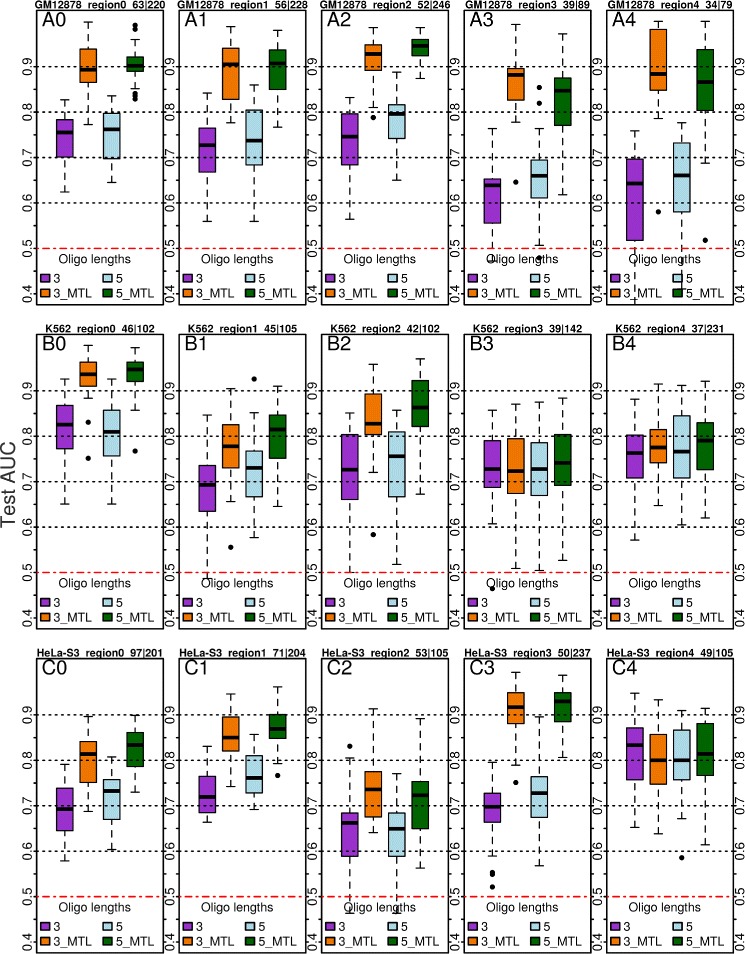
*Box-plots* of SVC performances for cell lines GM12878, K562 and Hela-S3. Five *regions* (numbered ‘A0-A4’, ‘B0-B4’ and ‘C0-C4’ for GM12878, K562 and Hela-S3 respectively) out of 10 are shown. Individual tasks setting, oligomer lengths = {3,5} in *purple* and *light blue* respectively. MTL with 10 tasks, oligomer lengths = {3,5} in *orange* and *green*. Distances between *K*-mer pairs upto *D*=100. *Box-plots* for the other five regions among the 10 are given in the Supplementary Figures S3, S4 and S5 in Additional file 1

The average test AUC values for the individual tasks are as follows. Oligomer length 3: {GM12878, K562, HeLa-S3}: {0.7251, 0.7534, 0.6782}; Oligomer length 5: {GM12878, K562, HeLa-S3}: {0.7443, 0.7716, 0.7153}. Box plots of all the test performances for different regions in all three cell lines are given in Fig. 2, and Additional file 1 (Supplementary Figures S3, S4 and S5 in Additional file 1). Owing to small sample sizes, the model test performances mostly show high variance (Fig. 2, and Supplementary Figures S3, S4 and S5 in Additional file 1).

For any interaction the complete length of the fragment may not be causal for the interaction, but only part(s) of it. However, this information is not available from the chromatin interaction experiments due to the length distribution of the fragments. Our locus-specific models are able to work around this situation and capture the features from different parts of the locus. This is due to the nature of ODH feature representations which capture the relative structure spread across the sequence rather than occurrences at different absolute positions in the sequence. Section “Tandem repeat motifs are an important feature distinguishing interaction partners” discusses how our visualizations help bring out this aspect of our models.

### Tandem repeat motifs are an important feature distinguishing interaction partners

**Fig. 3:**
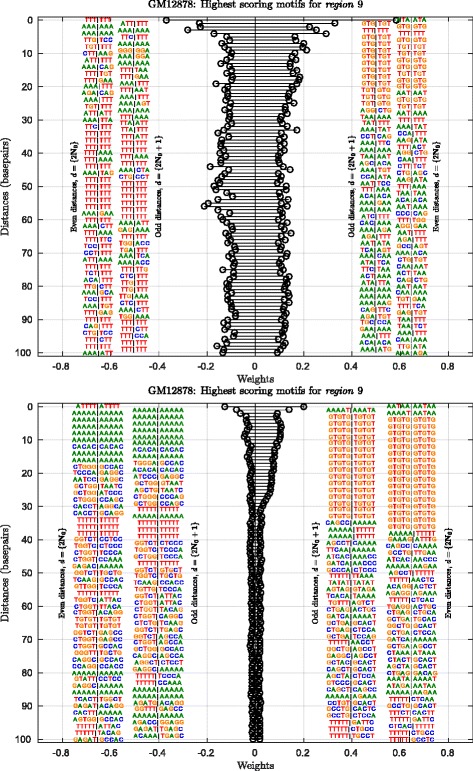
‘AMPD’ visualization of the informative *K*-mer pairs from the predictor for *region* 9 in GM12878 (Refer Table 1 for *region* details). *Top*: At distances in {0,…,100} basepairs, the 3-mer pair that maximally contributes towards positive and negative classification of a given locus is shown. Weights are shown on the *horizontal axis*, distances on the *vertical axis*. *Bottom*: ‘AMPD’ visualization for the 5-mer case

**Fig. 4:**
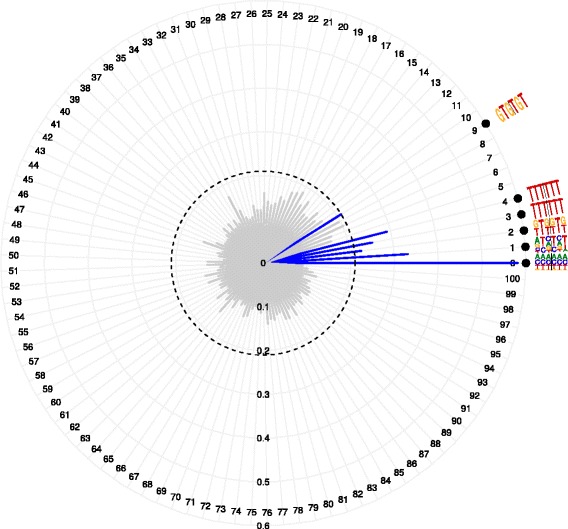
‘Top25’ visualization of the informative 3-mer pairs separated by various distances and their magnitudes from the predictor for *region* 9 in GM12878 (Refer Table 1 for *region* details). Top-25 3-mer pairs, with weight magnitudes higher than the threshold (*dashed inner circle*), for the positive class (*blue*). The *dashed inner circle* is the threshold to select the top-25 entries of the averaged SVM weight vector

Figures 3 and 4 show our new visualizations of the set of *K*-mer pairs that influenced the prediction most. In both these visualizations, any *K*-mer pair is represented as an adjoined { 2*K*}-mer separated by ‘ |’, e.g., 3-mer pairs as 6-mers, and we loosely address these *K*-mer pairs as ‘motifs’, although they are not contiguous. Figure 3 shows the ‘Absolute Max Per Distance’ (AMPD) visualization for a region (*region* 9) in cell line GM12878. The AMPD visualization shows, at each distance value (plotted on vertical axis), the *K*-mer pair that contributes the most in predicting a locus as positive and negative. The weights of these *K*-mer pairs (fetched from the SVM weight vector) are plotted on the horizontal axis. In the visualization, the *K*-mer pairs at even and odd distance values are segregated from each other to improve legibility. In the left panel, one sees 6-mers consisting of the 3-mer pairs separated by ‘ |’ (see Fig. 3), and in the right panel are 10-mers consisting of the 5-mer pairs. Owing to the high dimensionality of the 5-mer case, we observe that the magnitudes of the weights quickly shrink in this case. We filter this information further and visualize only the top few high-scoring features in the ‘TopN’ visualization shown in Fig. 4. At any distance value, all motifs that exceeded the threshold (shown as an inner dashed circle) are collected along with their weight magnitudes and stacked one over the other to finally represent them with a consensus motif (refer to “Visualizing the important features for each prediction model” section for more details). These consensus motifs are visualized radially.

Across various regions, among many motifs, tandem repeat sequences are prominently observed, especially di- and trinucleotide repeats, at various distances. Our ‘AMPD’ visualizations facilitate spotting of patterns spread over distances while the ‘TopN’ visualizations, due to the consensus motifs, can help spot possibly hidden shorter *K*-mer signals. Refer to Fig. 3 for the following discussion. The dinucleotide pattern ‘GT’ being repeated is observed in both cases, 3-mers and 5-mers, for distances up to 26 and 34 respectively, to have a maximal contribution among the various *K*-mer pairs towards predicting a locus as a potential interacting partner of locus chr21:34819525-34821921 (*region* 9) in GM12878. The 3-mer case shows patterns prominently containing more ‘T’s, from distance ∼30-60 as compared to the smaller distance values, among negatively contributing pairs, while the maximal positive contributors are devoid of them. Various such patterns are observed for different regions across cell lines.

Our literature search revealed some relevant studies on tandem repeat sequences and their potential biological roles. A 1990 review by Vogt [20] provides a very comprehensive and extensive account of the potential functions of tandem repeat sequences in the human genome [20]. Among many other things, it includes an exhaustive discussion of the various repeat sequences, viz. mono-, di-, tri-, tetranucleotides and beyond, and the postulates of their association with a multitude of nuclear proteins that help them assume specific chromosomal structures. The author terms this ability of the tandem sequence repeat blocks to render locus-specific higher order structure and play a role in organization as the ‘chromatin folding code’ [20]. In the review [20], the author also points to a specific case of the dinucleotide ‘TG’ as a simple repeating block, which has already been shown to have an enhancer function in vitro [21] in as early as 1984. More recently, a 2014 study [22] identified dinucleotide repeat motifs (DRMs) as general features that can render a nonfunctional sequence into an active enhancer element. Another comprehensive study of the simple sequence repeats in 2014 [23] suggests their potential role in genome regulation and organization. Variable number tandem repeats (VNTRs), as these sequence repeats are broadly termed, have already been implicated in many complex neurological disorders (e.g., Huntington disease [24]) and are generally known to be polymorphic [25].

With this backdrop, it is interesting that, enabled by the visualizations, our models using sequence-level information also reveal such tandem repeat motif signals (at times, even lengths of their tracts) as distinguishing characteristics between potential locus-specific interaction partners, suggesting a potentially important role of such sequence repeats in genome organization and regulation.

### Identifying cell-line specific characteristic signals among (non-)interactors of the same locus in different cell lines

**Fig. 5:**
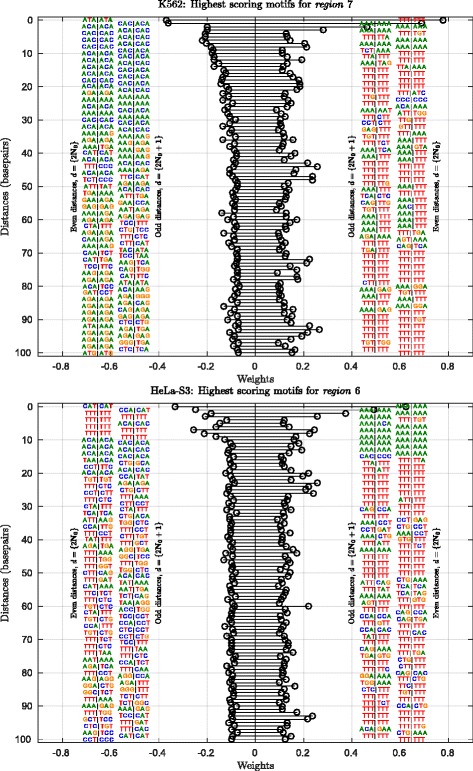
‘AMPD’ visualization of the informative 3-mer pairs from the classifiers for locus chr22:32170492-32188129 which is, both, *region* 7 in K562 and *region* 6 in HeLa-S3 (Refer Table 1 for *region* details). *Top panel*: At distances in (0-100) basepairs, the 3-mer pair that maximally contributes towards positive and negative classification of a given locus is shown. Weights are shown on the horizontal axis, distances on the vertical axis. *Bottom panel*: 3-mer ‘AMPD’ visualization of the same locus in HeLa-S3

As discussed in “Prediction of long-range chromatin interactions is possible from the sequence alone using non-linear SVMs” section, an advantage of studying locus-specific interactions at the sequence-level is realized when our models can reveal the characteristic signals among interaction partners of the same locus in two different cell lines. Consider the locus chr22:32170492-32188129 which is, both, *region* 6 and *region* 7 among our models for HeLa-S3 and K562 respectively (see Table 1). Refer to their ‘AMPD’ visualizations with 3-mers in Fig. 5. For K562, the ‘CA’ dinucleotide repeat sequence stretch of length ∼20 markedly denotes a non-interacting partner while this same repeat sequence seems to be interrupted with a short stretch of ‘T’s in HeLa-S3. Also, another repeat sequence, ‘AGA’, is notable beyond distance values 50 among the non-interacting partners for this locus in K562 as compared to HeLa-S3 where it is only intermittently observed. These signals are, similarly, also picked up by our 5-mer models. The 3-mer and 5-mer ‘AMPD’ visualizations for *region* 7 in cell line K562 and *region* 6 in HeLa-S3 are given in Supplementary Figures S9 and S12 respectively in Additional file 1. The corresponding ‘Top25’ visualizations for these regions are given in Supplementary Figures S10, S11, S13 and S14 in Additional file 1.

### Multitask learning (MTL) helps mitigate issue of having too few interacting partners per locus

Each locus-specific prediction problem in our scenario is termed as a *task* in the MTL setting. The small sample sizes in the single-task setting can be mitigated with the help of the so-called ‘multitask’ setting (see “Methodical details” for more details). In order to evaluate the efficacy of MTL for this problem, we used the available 10 individual tasks. Here, to compute the task similarity, we used the ‘model-defining’ locus (the LoI) information. The locus sequence of every ‘model-defining’ region was represented as an ODH feature vector using the *K*-mer values 3 and 5, separately, and maximum distance 100. The similarities between these regions, the *tasks*, were given by the resulting dot products. For models that used oligomer length 3 and 5 representations for the sample sequences, we used the corresponding task similarities also with oligomer length 3 and 5 respectively. The mean test AUC values for the multitask setting with 10 tasks are shown in columns marked ‘C’ and ‘D’ (oligomer length 3 and 5 respectively) of Table 1. Mean performance increase across all regions: Oligomer length 3: {GM12878, K562, HeLa-S3}: {0.13, 0.06, 0.13}; Oligomer length 5: {GM12878, K562, HeLa-S3}: {0.09, 0.06, 0.11}. Their box plots are shown in Fig. 2 and Supplementary Figures S3, S4 and S5 (Additional file 1). Performances in the MTL setting mostly show reduced variance as compared to the single-task performances.

Thus, our pipeline in the MTL setting can (a) mitigate the issue of having either too few interacting partners per locus, or (b) in the extreme case, identify putative interaction partners of a locus not profiled in the 5C experiment provided that at least some regions from the same cell line have been profiled in a chromatin interaction experiment, for example, 4C or 5C.

### Computational validation with high-resolution Hi-C

Rao et al. performed Hi-C experiments resulting in contact matrices at very high-resolution e.g. 1k, 5k, 10k, 25k base pairs (bps), etc. for various cell lines including GM12878, K562 [10]. Corresponding to the ‘model-defining’ *regions*, we picked relevant columns from the 5k Hi-C *cis*-contact matrix of the relevant chromosome. For example, if the ‘model-defining’ genomic *region* was 12,000 bps long, we collected candidate regions (across the rows) corresponding to three column loci. The candidate regions are those which have a non-zero KR-normalized [26] interaction frequency with the LoI. After normalizing, to identify significantly interacting partners at any given resolution, we computed their observed/expected (O/E) values and used an ad-hoc cutoff of 2.5 (i.e., a locus with a normalized O/E value ≥ 2.5 was considered significantly interacting with the LoI), as used earlier in [9]. This criterion is made more stringent as follows. The final set of loci that are considered significantly interacting with any individual ‘model-defining’ *region* are only those that are significant at 5k resolution and also at 10k or 25k resolutions (all using the same cutoff). In other words, if a locus was deemed significant only at 5k resolution but not at 10 or 25k, then we did not consider it a true positive.

These *cis*-interacting genomic loci from the high-resolution contact maps are treated as unseen test sequences for the classifiers built for each *region* using the 5C data. In the pipeline, these are thus treated similarly to the 20% hold-out set: their ODH feature representations are fed to the classifier to predict their labels. We performed this experiment for cell lines GM12878 and K562.

When evaluating performances of our models regarding predictions on unseen loci from Hi-C data, we did so for two scenarios: (a) all chromosome-wide loci together; and (b) considering only loci lying beyond 1M bps from the ‘model-defining’ locus, i.e., excluding the regions probed in the 5C experiment [18] for the evaluation. Using this stringent criterion, the mean AUC values and their standard deviations are as follows. For prediction with oligomer length 3 models (a) chromosome-wide interaction partners: {GM12878, K562} : { 0.5358±0.025, 0.5122±0.084}; (b) interaction partners beyond 1M bps: { 0.5327±0.019, 0.5304±0.057}. And, with oligomer length 5 models (a) chromosome-wide interaction partners: {GM12878, K562} : { 0.5278±0.028, 0.5238±0.081}; (b) interaction partners beyond 1M bps: { 0.5220±0.026, 0.5294±0.064}. For both cell lines, when considering only the first five regions, the average performance was ∼0.55 test AUC (see Table 2). Models for K562 show higher variance than models for GM12878.

**Table 2:** Computational validation with high-resolution Hi-C data

Cell-type	Oligomer length 3 (mean ±s.d.)	Oligomer length 5 (mean ±s.d.)
Chromosome-wide interaction partners		
GM12878 (regions 0-4)	0.5552±0.009	0.5503±0.006
GM12878 (regions 0-9)	0.5358±0.025	0.5279±0.028
K562 (regions 0-4)	0.5508±0.091	0.5650±0.088
K562 (regions 0-9)	0.5122±0.084	0.5239±0.081
Interaction partners beyond 1M bp		
GM12878 (regions 0-4)	0.5468±0.005	0.5419±0.007
GM12878 (regions 0-9)	0.5327±0.019	0.5220±0.026
K562 (regions 0-4)	0.5593±0.062	0.5646±0.064
K562 (regions 0-9)	0.5304±0.058	0.5294±0.064

(s.d.: standard deviation)

We observed that performances of models predicting interaction partners for some LoI are comparatively poorer than those of other models. These ‘model-defining’ LoI either have very few negative samples to learn from (refer to Table 1) or are themselves rather long loci (refer to column ‘length (bp)’ in Supplementary Table S1 in Additional file 1). In general, from the perspective of training on 5C data and predicting contacts chromosome-wide, the issues of having few negative samples to learn from and having a rather long model-defining *region* (both, in 5C data) make the problem harder. This could be due to following reasons: (a) the experiments give no information on the potential causal portion(s)(causal for the said interaction), if any, along the complete restriction fragment; (b) the interacting as well as non-interacting partners of a rather long ‘model-defining’ locus could have varying characteristics in them which may not be comprehensively captured by the available few samples in the 5C data; and (c) the 5C experiments are performed on selected promoter regions and distal enhancers [18] while we make these models trained on such restricted 5C data to predict a potential interaction partner anywhere on the genome not just promoter or distal enhancer regions. Thus, learning on 5C data for a very small subset of the chromosome and then predicting interactions chromosome-wide is a very hard problem (see for example [14], and “Related work” section).

### Related work

Recently, Roy et al. [14] developed a model for predicting cell-line specific interactions between only enhancers and promoters using various regulatory genomic datasets. Their predictive model learns from interacting and non-interacting pairs, also from 5C data [18], where the participating promoter and enhancer (of a contact-pair) are encoded as a real or binary vector marking information from 23 datasets including histone marks and transcription factor binding for various cell lines. Additionally, they also attempt at building a minimal classifier that uses information from 11 datasets out of the 23. They achieved a performance (area under precision-recall curve (auPRC)) of ∼0.75-0.78 when training and predicting on the same experiment (5C) data. They also performed tasks of training on 5C data [18] and predicting interactions in high-resolution Hi-C data [10]. For this task, they consider an interaction involving a 5k bps locus pair as a true interaction if it is called a peak in any one of the three resolutions 5k, 10k and 25k, and achieved comparatively modest performances (auPRCs) of 0.643 (K562) and 0.687 (GM12878).

In comparison to the literature for prediction of promoter-enhancer interactions, we have used the term long-range chromatin interactions in a broader sense that includes possible interactions between intervening chromatin regions in addition to those (significant looping interactions) between specific genomic (functional) elements such as the enhancers and promoters. We hypothesize that the intervening chromatin could play an important role in maintaining a favorable landscape for the loci to interact, as also observed in more recent capture-C experiments data [27], where there is a possibility of weaker interactions due to putative low-affinity binding sites (e.g., [28]) which, in general, have been largely unexplored still. In our work we have focused on characterizing the long-range chromatin interactions pertaining to a particular genomic locus and investigating the capability of genomic sequence alone in characterizing them. Also, for the task of learning on 5C data and predicting on high-resolution Hi-C data, we have used a comparatively more stringent criterion for considering an interaction a true one. Approaches that use various additional information sources, e.g., epigenetic information [14], typically leave out genomic regions for which these are not available. Our sequence-based approach can be especially helpful in such scenarios. Furthermore, we expect that our models can be further strengthened or supported by utilizing the additional regulatory (epi)genomic information wherever available.

## Conclusion

To the best of our knowledge, from the point of view of understanding chromatin interactions at the sequence level, ours is the first approach to do so. In this study, we have taken a broader view of these interactions and based on the hypothesis that the sequence at the intervening chromatin and the loci could also play a part in these interactions given the possibility of such ‘interfacing’ taking place via various mechanisms, like direct contact or formation of mini-loops or via diffusion after mere juxtaposing in physical vicinity [2], and for various reasons as motivated in the “Background” section. Our computational experiments using data from 5C experiments, for three cell lines GM12878, K562 and HeLa-S3 from [18] achieve good performances of ∼0.75 (with oligomer length 5, as average test AUC values across various regions evaluated in this study from the three cell lines) in the single-task setting.

We developed two new, intuitive visualization methods that are suited for our problem scenario namely dealing with varied-length sequences and an appropriately chosen ODH feature representation. Aided by these visualizations, notwithstanding the very high-dimensionality of the feature space (e.g., the 5-mer case), our per-locus models shed light on the potential sequence signals that can characterize the interacting vs. the non-interacting partners of a LoI. We discussed how this can help understand which sequence features in the given region made it interact with one LoI and not with another LoI. Analysis of the various sequence signals from our models suggests a potential functional and organizational role for tandem repeat sequence stretches in the genome.

We also demonstrated how knowledge of individual models could be transferred to those of other regions (those having too few examples to learn from) via multitask learning. Mean performance for the multitask setting, performances of models for oligomer length 3 and 5 combined together, is 0.83. We already observed that several models show less variance in their prediction performances than their single-task counterparts.

Furthermore, we made our models trained on 5C data predict interactions between 5k bps long loci from the recent high-resolution Hi-C [10] data for cell lines where the Hi-C data was available. Even with a very stringent criterion to identify true positives in the high-resolution Hi-C data, we showed that our approach is capable of predicting interesting loci that could interact although lying very far away, even further than 1–2M bps, on the genome using features learned from 5C data that is limited to this 1–2M bps distance. This ability to identify potentially interacting loci lying very far away on the genome could be useful from the point of view of understanding topologically associating domains at the sequence level.

An important point to note here is that since our models do not require any locus to be either a TCR or an enhancer region per se, in principle, it can be seamlessly applied to contact matrices output by any 5C-based or even high resolution Hi-C-based experiments (as training data). At places, we have used the terms TCR and enhancers for the interacting regions because the contact matrices we use in this study come from 5C experiments involving these loci. So, when given a Hi-C contact matrix, any locus therein could be used to learn corresponding models in a similar fashion and it need not necessarily be an enhancer or a promoter region. In comparison, earlier approaches focus only on promoter-enhancer interactions and exclude all other genomic loci from their analysis. Thus, we have preferred to call these genomic loci as simply *regions* in this study. The models in this work are not specific to particular properties of any genomic region and do not make use of supplementary epigenetic information at the locus; we have only used the sequence information. Even with this much harder premise, we still achieved a good performance of ∼0.75.

As of today, high resolution Hi-C data is still very expensive. Therefore, our prediction method could also be used in a setting where high-resolution 5C data, but only low-resolution Hi-C data is available to predict additional interaction partners for any regions of interest. These additional predicted contacts could augment methods for predicting the 3D structure of the chromatin as well as methods for predicting boundaries of TADs. Thus, we envisage that our approach of using only sequence-based models can, most importantly, be helpful in (a) understanding higher-order structures like (meta-) TADs at the sequence-level; and (b) giving additional input to methods that estimate the 3D structure of the chromatin for different organisms from the interaction data.

## Methods

### Materials

We use the 5C contact matrices from experiments published by Sanyal et al. [18]. They probed a collection of regions for two tier-I cell lines (GM12878 and K562) and a tier-II cell line (HeLa-S3) from ENCODE (The ENCODE Project Consortium, 2012). In these experiments involving two biological replicates, for each replicate, upon filtering to exclude certain primers owing to outlier fragments, the contact frequencies are normalized for the trans signal in turn correcting for detection biases per restriction fragment [18]. The intra-chromosomally interacting restriction fragments are then tested for significance, accounting for the inverse relationship between contact frequencies and the genomic distance between the restriction fragments, and peaks are called, conservatively, at a false discovery rate (FDR) cutoff of 1%. [18] term the interactions that are called peaks in both replicates as ‘TruePeaks’ and those not called peaks in either replicate as ‘NonPeaks’. Consequently, in our study, positive examples for any classifier are ‘TruePeaks’ and negative examples, ‘NonPeaks’. We considered different FDR cutoff values (1%, 10% and 15%) and selected an FDR cutoff of 10% (discussed in “Relaxation of FDR cutoff to enable studying of putative ‘bystander’ or structural interactions” section). Table 1 gives information on the number of ‘TruePeaks’ (#TP) and the number of ‘NonPeaks’ (#NP) for the genomic regions for which we built our models in this study to evaluate whether the DNA sequence is informative in predicting the long-range interactions (Refer to Supplementary Tables S1–S4 for additional details about the studied genomic regions). These are the ‘model-defining’ *regions* for our study. All genomic coordinates are w.r.t. hg19, GRCh37 assembly. The ‘model-defining’ loci are among the TSS-containing regions (by GENCODE v7 [29]) and the sets of loci in the positive and negative class for the individual classifiers are restriction fragments corresponding to enhancers (also by GENCODE v7 [29]) [18]. All values of #TruePeaks and #NonPeaks in Table 1 are for FDR 10%. For the computational validation with high-resolution Hi-C data, we used the data from Rao et al. [10] deposited at GEO [30], namely ‘GSE63525_GM12878_combined_contact_matrices.tar.gz’ and ‘GSE63525_K562_intrachromosomal_contact_matrices.tar.gz’.

#### Relaxation of FDR cutoff to enable studying of putative ‘bystander’ or structural interactions

From a biological point of view, we attempted to take a more broader view and defined an interaction that takes into account not just the significant ‘looping interactions’ but also the possibility of so-called ‘bystander’ or structural interactions between intervening chromatin [18, 27]. Thus, in all computational experiments, in order to distinguish significant interactions from non-interactions in the 5C data, we relaxed the FDR cutoff to 10%, instead of 1% as in [18]. In other words, we traded off between being very conservative (which would allow only significant ‘looping interactions’ as prevalently defined in the community) and comparatively liberal in considering TruePeaks at FDR cutoff 10%. At the same time, this relaxation still maintained a significantly higher mean z-score of the interactions for TruePeaks in comparison to NonPeaks for all the cell lines, similar to the 1% cutoff case (see Supplementary Figure S1 in Additional file 1). While, 15% FDR also shows a significant difference, it did not provide much benefit in the number of additional TruePeaks per region (i.e., positive examples per classification problem in our study) in comparison to relaxing the FDR from 1 to 10%, consistently across all three cell lines.

### Methodical details

The genomic loci we study in this work are the restriction fragments reported in the 5C experiments in [18] (see “Materials” section for details). We use string kernels, which provide a measure of similarity between sequences, in conjunction with an SVM as a classifier. Because these loci have highly diverse lengths (Supplementary Figure S2 in Additional file 1), we could not directly use position-aware string kernels like the oligo kernel [31] or weighted degree (WD) kernels [32, 33] for representing the loci.

#### A feature representation based on oligomer distance histograms (ODH) and the ODH kernel

In 2006 Lingner and Meinicke introduced the ODH feature representation and the corresponding ODH kernel [19]. It provides a fixed-length feature space representation of any arbitrary length sequence based on histograms of distances between short oligomers in the sequence. For alphabet $\sum $, consider all oligomers (or interchangeably, *K*-mers) $m_{i} \in \sum ^{K}, i = 1,\ldots,M$. For any sequence s of length |s|:=*L*_max_, let *D*=*L*_max_−*K*, the maximum distance between any two *K*-mers, with distance between a pair of *K*-mers defined as the difference in their starting positions in the sequence s. The distance histogram vector of s corresponding to the *K*-mer pair (*i,j*) is given by ${\mathbf{h}}_{ij}(\mathrm {s}) = [h_{ij}^{0}(\mathrm {s}), h_{ij}^{1}(\mathrm {s}), \ldots, h_{ij}^{D}(\mathrm {s})]^{\mathrm {T}}$ where T denotes transpose. For all such *K*-mer pairs over $\sum $, the corresponding distance histogram vectors are concatenated together giving a complete feature space transformation *Φ*(s).

1 $$\begin{array}{@{}rcl@{}} \Phi(\mathrm{s}) = \left[{\mathbf{h}}_{11}^{\mathrm{T}}(\mathrm{s}), {\mathbf{h}}_{12}^{\mathrm{T}}(\mathrm{s}), \ldots, {\mathbf{h}}_{MM}^{\mathrm{T}}(\mathrm{s})\right]^{\mathrm{T}} \end{array} $$

The set of feature vectors for *N* training samples is: **X**=[*Φ*(s_1_),…,*Φ*(s_*N*_)] and the *N*×*N* kernel matrix is given by:

2 $$\begin{array}{@{}rcl@{}} \mathbf{K} = \mathbf{X}^{\mathrm{T}}\mathbf{X} \end{array} $$

with *k*_*ij*_, the entries of matrix **K**, being proportional to the similarity between sequence s_*i*_ and s_*j*_. Lingner and Meinicke used this kernel for remote homology detection in protein sequences [19].

#### Multitask learning (MTL)

Often, for various reasons across domains, one has to deal with the issue of having very few training samples for a given prediction problem also called task. This can affect the generalization ability of any standard machine learning technique such as an SVM [34]. When multiple related tasks are to be learnt, MTL attempts to mitigate this issue by sharing information across these multiple related tasks. From a different perspective, it can be advantageous to leverage information from multiple related tasks to improve the prediction performance of a single task [34]. Depending upon the problem at hand, a suitable measure of task-relatedness (how similar are two given tasks) needs to be chosen.

In case of learning with kernels, [35] introduced how multitask learning can be performed with kernel methods. Jacob and Vert [36] provided the following formulation for sharing of information between tasks using a multitask kernel. For any two samples *s*_*A*_ and *s*_*B*_ from tasks *t*_*A*_ and *t*_*B*_ respectively, *K*_*MTL*_((*s*_*A*_,*t*_*A*_),(*s*_*B*_,*t*_*B*_)) is the multitask kernel providing a measure of similarity between these tuples. Mathematically, *K*_*MTL*_((*s*_*A*_,*t*_*A*_),(*s*_*B*_,*t*_*B*_))=*K*_*S*_(*s*_*A*_,*s*_*B*_)·*K*_*T*_(*t*_*A*_,*t*_*B*_) where *K*_*S*_ is the kernel on the samples, and *K*_*T*_ gives the kernel value between two tasks. Jacob and Vert [36] used this formulation for predicting peptide–MHC-I binding. An overview of MTL applications for problems in computational biology is presented by [37].

#### Pipeline for predicting long-range chromatin interactions

Contact matrix output by any experiment profiling chromatin interactions must be subjected to normalization and extraction of significant contacts. Details of the motivation and various approaches for doing so are reviewed Ay and Noble [38]. Also, these experiments are usually performed for multiple biological replicates to assess the impact of experimental errors and other variations.

Figure 1 illustrates our approach for predicting long-range chromatin interactions. The normalization and peak-calling procedures that we adopted for analyzing the 5C data used in this study are described in “Materials” section. Once a raw contact matrix has been normalized and the significant interactions have been called, we binarize the contact matrix as follows. Genomic loci (along the rows) not called significant interaction partners of a particular locus (along the columns) in either replicate constitute the negative class (see Fig. 1, cells denoted by filled black boxes) and those called significant in all replicates constitute the positive class (see Fig. 1, cells denoted by filled orange boxes). This leaves a lot of uncalled loci (along the rows). These are denoted by unfilled boxes (Fig. 1). Then, we build a classifier corresponding to each locus along the column of the matrix. We call these loci the ‘model-defining’ loci. For each individual classifier we collect loci along the rows and falling under the relevant column of the contact matrix as loci belonging to the positive and negative class for this classifier or it may not be called at all. This is shown in Fig. 1. Clearly, any locus that belongs to the positive class in one model, may belong to either the positive or negative class in another model. Given a set of sequences belonging to either class, 80% were used for training a classifier while 20% were held-out as test sequences.

The classifiers are based on SVMs with the ODH kernel. The cost parameter for each SVM is varied in the range 10^{−3,…,3}^. For each model, we perform a 5-fold nested cross-validation to select the best performing SVM cost-value while the ODH feature representation parameters are fixed as described in “Prediction of long-range chromatin interactions is possible from the sequence alone using non-linear SVMs” section. Our pipeline also accounts for class-imbalance by proportionately up-weighting the misclassification cost for the minority class (here, positive class) [39].

Our pipeline, named ‘Samarth’, is available for download at the supplemental website http://bioinf.mpi-inf.mpg.de/publications/samarth/.

#### Visualizing the important features for each prediction model

*Absolute Max Per Distance (AMPD) visualizations*: Recall from “A feature representation based on oligomer distance histograms (ODH) and the ODH kernel” section that the dimensionality of the SVM weight vector for a model with the DNA sequence alphabet, using oligomer length *K* and distances up to *D* is $[\!(|\sum |^{K})^{2} \times (D + 1)]$ (i.e., of 413,696 and 105,906,176 dimensions for oligomer length 3 and 5 respectively). Due to the oligomer distance histograms-based feature vector representation used in our models, each entry of the SVM weight vector is the coefficient assigned to a *K*-mer pair separated by a distance *d*∈ [ 0,1,…,*D*]. For each of our locus-specific models, the 5-fold outer cross validation resulted in 5 different SVM weight vectors. These five individual weight vectors were averaged to obtain one representative weight vector for a per-locus model. From this averaged weight vector, we noted two *K*-mer pairs per distance value, one that was assigned the most positive coefficient and the other, most negative. A positive coefficient means the *d*-separated *K*-mer pair is an important feature among the positive sequences, while a negative coefficient means it is an important feature to classify the sequence as negative. All such selected *K*-mers at the various distance values are visualized to provide a distance-centric view of the important features. Such a visualization for an example *region* (*region* 9) for cell line GM12878 is shown in Fig. 3. We call these visualizations ‘Absolute Max Per Distance’ (AMPD) visualizations. For better readability, the *K*-mer pairs at even distance values are arranged in the outer column and those at odd distance values in the inner column. Figure 3 and Supplementary Figure S6, S9 and S12 in the Additional file 1 show examples of ‘AMPD’ visualizations for different regions across the three cell lines GM12878, K562 and HeLa-S3.

*Position-Wise Weight Matrix (PWWM)-based ‘TopN’ visualizations*: Independently, the entries of the averaged weight vector were sorted in descending order and then thresholded to reveal the top 25 scoring entries. Figure 4 visualizes only those selected top-25 *K*-mer pairs. Here, the (*D*+1) distances are arranged radially. Each spoke gives the magnitude of the highest-scoring *K*-mer pair at the corresponding distance. If the magnitude crosses the threshold value, that spoke is plotted in either ‘blue’ (see Fig. 4) or ‘red’ (see Additional file 1) for positive and negative contribution respectively, while otherwise plotted in gray. We call these visualizations ‘Top25’, or more generally, ‘TopN’ visualizations where one can choose a suitable value for ‘N’. Note that there can be several entries at the same distance among the top-25 leading to sequence logo-like representations. At any distance *d*, all motifs that exceeded the threshold are collected along with their weight magnitudes and stacked one over the other to finally represent them with a consensus motif. This is done by constructing a ‘Position-Wise Weight Matrix’ (PWWM) of dimension $(|\sum | \times 2\mathit {K})$ which represents the nucleotides appearing at each position from 1 to 2*K* along with their relative contribution to the weight vector. A dummy example illustrating this is shown in Table 3. This PWWM is computed as follows. For position *p*∈{1,…,2*K*}, the matrix cell (‘A’/‘C’/‘G’/‘T’, *p*) is populated with the sum of the weight contribution of those motifs in which the given nucleotide is present at position *p*. The matrix is then normalized for the column entries to sum up to 1. The resulting consensus sequences are represented as sequence logos [40] in the ‘Top25’ visualizations in Fig. 4. Supplementary Figures S7, S8, S10, S11, S13 and S14 in the Additional file 1 show example ‘Top25’ visualizations for various *regions* from the cell lines GM12878, K562 and HeLa-S3.

**Table 3:** A dummy PWWM for selected 3-mer pairs at certain distance *d*. |*w*_1_|, |*w*_2_|, and |*w*_3_| are magnitudes of the weights for the example 3-mer pairs

	3-mer pairs					
|*w*_1_|	AAA			GAA		
|*w*_2_|	GAA			AGA		
|*w*_3_|	AAG			AAA		
‘A’	$\frac {1}{D}(|w_{1}|+|w_{3}|)$	$\frac {1}{D}(|w_{1}|+|w_{2}|+|w_{3}|)$	$\frac {1}{D}(|w_{1}| +|w_{2}|)$	$\frac {1}{D}(|w_{2}|+|w_{3}|)$	$\frac {1}{D}(|w_{1}|+|w_{3}|)$	$\frac {1}{D}(|w_{1}|+|w_{2}|+|w_{3}|)$
‘C’	0	0	0	0	0	0
‘G’	$\frac {1}{D}(|w_{2}|)$	0	$\frac {1}{D}(|w_{3})$	$\frac {1}{D}(|w_{1}|)$	$\frac {1}{D}(|w_{2})$	0
‘T’	0	0	0	0	0	0
*p*	1	2	3	4	5	6

‘A’, ‘C’, ‘G’ and ‘T’ are the rows corresponding to the nucleotides. Position, *p*∈{1,…,6}. Each cell is divided by *D*=(|*w*_1_|+|*w*_2_|+|*w*_3_|)

## Additional file

Additional file 1 This file provides additional performance plots and visualizations, and more detailed description of the data. Figure S1: Z-scores for various cell lines at different FDRs Figure S2: Lengths of restriction fragments for various regions in different cell lines Figure S3: Box-plots of SVC performances for all regions (numbered ‘A0-A9’) in GM12878 Figure S4: Box-plots of SVC performances for all regions (numbered ‘B0-B9’) in K562 Figure S5: Box-plots of SVC performances for all regions (numbered ‘C0-C9’) in HeLa-S3 Figure S6: ‘AMPD’ visualization of the informative K-mer pairs from the classifier for region 9 in GM12878 Figure S7: ‘Top25’ visualization of the informative 3-mer pairs separated by various distances and their magnitudes from the classifier for region 7 and 9 in GM12878 Figure S8: ‘Top25’ visualization of the informative 3-mer pairs separated by various distances and their magnitudes from the classifier for region 7 and 9 in GM12878 Figure S9: ‘AMPD’ visualization of the informative K-mer pairs from the classifier for region 7 in K562 Figure S10: ‘Top25’ visualization of the informative 3-mer pairs separated by various distances and their magnitudes from the classifier for region 7 in K562 Figure S11: ‘Top25’ visualization of the informative 3-mer pairs separated by various distances and their magnitudes from the classifier for region 7 in K562 Figure S12: ‘AMPD’ visualization of the informative K-mer pairs from the classifier for region 6 in HeLa Figure S13: ‘Top25’ visualization of the informative 3-mer pairs separated by various distances and their magnitudes from the classifier for region 6 in HeLa Figure S14: ‘Top25’ visualization of the informative 3-mer pairs separated by various distances and their magnitudes from the classifier for region 6 in HeLa Table S1: Details of the genomic regions from each cell line Table S2: Overlap of candidate loci among regions for cell line GM12878 Table S3: Overlap of candidate loci among regions for cell line K562 Table S4: Overlap of candidate loci among regions for cell line HeLa. (PDF 1086 kb)

## Abbreviations

AMPD: Absolute max per distance
AUC: Area Under the receiver operating characteristic (ROC) Curve
auPRC: Area under precision-recall curve
bp: Base pairs
DRMs: Dinucleotide repeat motifs
FDR: False discovery rate
kb: kilobases
LoI: Locus of interest
MTL: Multitask learning
ODH: Oligomer distance histograms
PWWM: Position-wise weight matrix
SVM: Support vector machine
TCR: TSS-containing region
ToI: TCR of interest
TSS: Transcription start site
VNTRs: Variable number tandem repeats
