# Supplementary material for: Genetic sequence-based prediction of long-range chromatin interactions suggests a potential role of short tandem repeat sequences in genome organization
Source: BMC Bioinformatics. 2017 Apr 18;18:218. doi: 10.1186/s12859-017-1624-x (PMC5395875; doi:10.1186/s12859-017-1624-x)
Supplement: Additional file 1 — This file provides additional performance plots and visualizations, and more detailed description of the data. Figure S1: Z-scores for various cell lines at different FDRs Figure S2: Lengths of restriction fragments for various regions in different cell lines Figure S3: Box-plots of SVC performances for all regions (numbered ‘A0-A9’) in GM12878 Figure S4: Box-plots of SVC performances for all regions (numbered ‘B0-B9’) in K562 Figure S5: Box-plots of SVC performances for all regions (numbered ‘C0-C9’) in HeLa-S3 Figure S6: ‘AMPD’ visualization of the informative K-mer pairs from the classifier for region 9 in GM12878 Figure S7: ‘Top25’ visualization of the informative 3-mer pairs separated by various distances and their magnitudes from the classifier for region 7 and 9 in GM12878 Figure S8: ‘Top25’ visualization of the informative 3-mer pairs separated by various distances and their magnitudes from the classifier for region 7 and 9 in GM12878 Figure S9: ‘AMPD’ visualization of the informative K-mer pairs from the classifier for region 7 in K562 Figure S10: ‘Top25’ visualization of the informative 3-mer pairs separated by various distances and their magnitudes from the classifier for region 7 in K562 Figure S11: ‘Top25’ visualization of the informative 3-mer pairs separated by various distances and their magnitudes from the classifier for region 7 in K562 Figure S12: ‘AMPD’ visualization of the informative K-mer pairs from the classifier for region 6 in HeLa Figure S13: ‘Top25’ visualization of the informative 3-mer pairs separated by various distances and their magnitudes from the classifier for region 6 in HeLa Figure S14: ‘Top25’ visualization of the informative 3-mer pairs separated by various distances and their magnitudes from the classifier for region 6 in HeLa Table S1: Details of the genomic regions from each cell line Table S2: Overlap of candidate loci among regions for cell line GM12878 Table S3: Overlap of candidate loci among regions for cell line K562 [file 12859_2017_1624_MOESM1_ESM.pdf]

Supplementary File for ‘Genetic sequence-based prediction of long-range chromatin interactions suggests a potential role of tandem repeat sequences in genome organization’

Sarvesh Nikumbh and Nico Pfeifer
Department of Computational Biology and Applied Algorithmics,
Max Planck Institute for Informatics, Germany

Preparation of data

Here, Supplementary Figure S1 is an extension of the Supplementary Figure 1 from Sanyal et al. [1]. In this study we work with FDR 10% (refer to subsection “Relaxation of FDR cutoff to enable studying of putative ‘bystander’ or structural interactions” in main text). We have followed the

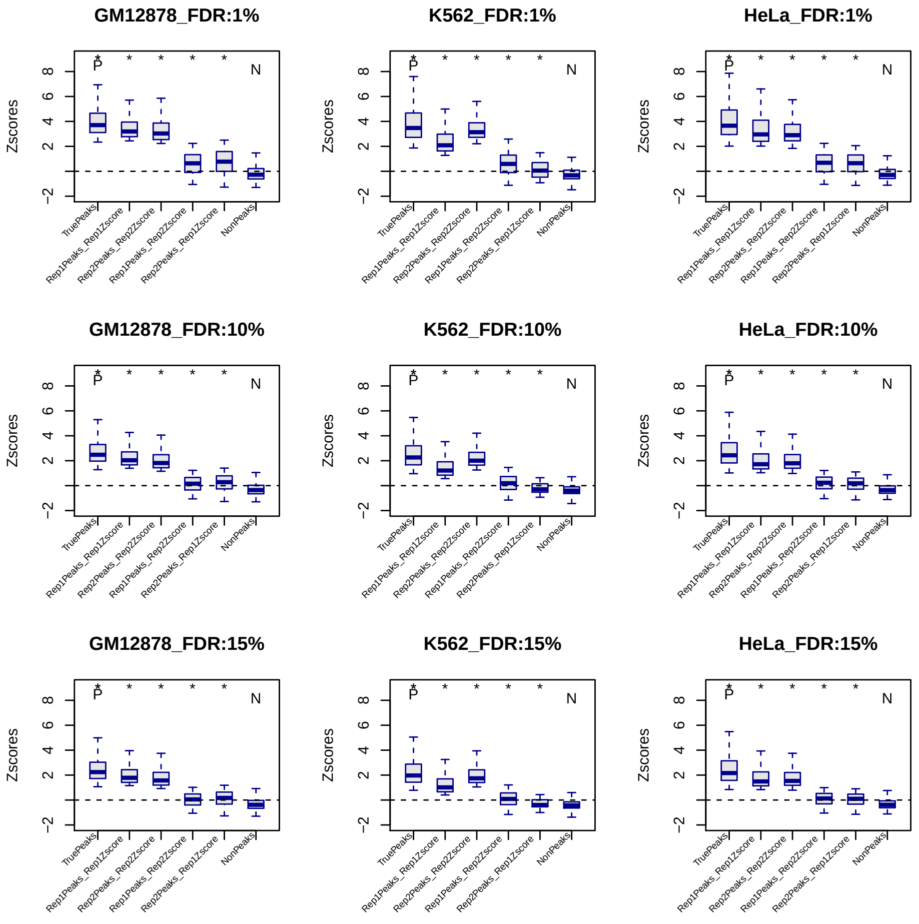

Figure S1: Z-scores for various cell lines at different FDRs. Refer to text for further explanation.

nomenclature from Sanyal et al. [1]. Rep1Peak_Rep1Zscore: peak in rep1, z-score in rep1 plotted; Rep2Peak_Rep2Zscore: peak in rep2, z-score in rep2 plotted; Rep1Peak_Rep2Zscore: peak in rep1,

z-score in rep2 plotted, Rep2Peak_Rep1Zscore: peak in rep2, z-score in rep1 plotted; TruePeaks: called peak in both replicates; NonPeaks: not called peak in either replicate. We compared each z-score distribution of the different peak classes to the z-score distribution of the NonPeaks with an unpaired Wilcoxon test. Asterisks (*) are shown for significant difference in z-score distribution at significance level 0.05. Note that we did not correct for multiple testing to keep the analysis comparable to Sanyal et al. [1]. The marks 'P' and 'N' on the box-plots for TruePeaks and NonPeaks denote they constituted the positive- and negative-set of examples respectively in our work.

Details of *regions*

For the three cell lines GM12878, K562 and HeLa-S3 from the 5C experiments data performed by Sanyal et al. [1], details of the genomic regions that defined the models in our case are given in Table S1. All genomic coordinates are w.r.t. hg19, GRCh37 assembly. These are among the TSS-containing regions (according to the GENCODE v7 [2]) for which reverse 5C primers were designed by Sanyal et al. [1].

The TCRs defined the models while the distal enhancers were grouped into two classes–those significantly interacting (peaks in both replicates with FDR 10 %–'TruePeaks') and those not interacting (non-peak in either replicate–'NonPeaks') with the TCR of interest. The genomic regions were ordered by the number of 'TruePeaks' for the regions from highest to lowest and the *regions* shown above are the top-10 among them. The restriction fragments that qualified as 'TruePeaks' are considered as positive examples and 'NonPeaks' as negative for the model corresponding to the given genomic region.

As discussed in the main text, our models can be built to study interactions involving any given genomic region which does not necessarily have to be a TSS-containing region or a distal enhancer. We use 'TCR' and 'distal fragments' since the 5C experiments specifically study these regions. For more, refer to section "Conclusion" in the main text.

Overlap of candidate loci of different *regions* in the three cell lines

Tables S2, S3 and S4 show the overlap in the set of candidate loci for different *regions* in all the three cell lines. A locus would fall in the intersection of two *regions* if it is interacting with both the *regions* of interest or not interacting w.r.t. both. Since we are working with intra-chromosomal interacting pairs, for cell line GM12878: *regions* 3, 4 (Chr5) have no overlap with either of *regions* 0, 1 and 2 (Chr7). Similarly for cell lines K562: *regions* 3 (Chr21) or 4 (Chr7) have no overlap with *regions* 0, 1 and 2 (Chr22) or between themselves and for HeLa-S3: *region* 2 (Chr22) shows no overlap with any of the other *regions* (Chr7) (refer Table S1). This clearly indicates that some sequences which are negative (non-interacting) w.r.t. a given TCR are positive w.r.t. another and vice-versa.

Lengths of restriction fragments for various *regions* in different cell lines

Figure S2 gives information on the lengths of the restriction fragments that are considered either positive or negative for the corresponding genomic *region* defining the model.

Implementation

For the ODH feature representation and kernel computation, we adapted the MATLAB code made available by authors of [3] for our purposes. Their implementation needed adaptations in order to handle very long sequences (refer Figure S2) which rendered the feature vectors non sparse.

We used LIBSVM [4] for the SVM implementation. Our complete pipeline with all the wrappers, and the additional MTL implementation is written in MATLAB.

[1] TCR locus lengths in base pairs (bp).

Table S1: Details of the genomic *regions* from each cell line.

**GM12878**						
region	TCR	length (bp[1])	TruePeaks	Rep1Peaks	Rep2Peaks	NonPeaks
0	chr7:115847372-115857098	9727	63	120	116	226
1	chr7:115890993-115892266	1274	56	124	97	234
2	chr7:115861595-115870968	9374	52	88	111	252
3	chr5:131722317-131724751	2435	39	53	50	91
4	chr5:131892428-131895867	3440	34	52	57	80
5	chr7:90224881-90229046	4166	34	51	97	122
6	chr7:116434729-116454408	19680	33	63	77	292
7	chr7:90337078-90341001	3924	32	67	43	158
8	chr22:32162110-32166713	4604	31	74	52	127
9	chr21:34819525-34821921	2397	30	48	44	201
**K562**						
region	TCR	length (bp)	TruePeaks	Rep1Peaks	Rep2Peaks	NonPeaks
0	chr22:32764253-32784733	20481	46	101	62	105
1	chr22:32920308-32927723	7416	45	101	57	109
2	chr22:32012966-32043914	30949	42	77	83	104
3	chr21:35242603-35256847	14245	39	100	52	150
4	chr7:115847372-115857098	9727	37	125	73	238
5	chr7:89787744-89795672	7929	35	97	56	118
6	chrX:153625659-153635385	9727	34	55	43	46
7	chr22:32170492-32188129	17638	32	83	74	97
8	chr22:32740683-32750950	10268	32	85	57	112
9	chr11:5721056-5732713	11658	31	67	40	85
**HeLa-S3**						
region	TCR	length (bp)	TruePeaks	Rep1Peaks	Rep2Peaks	NonPeaks
0	chr7:115847372-115857098	9727	98	152	138	207
1	chr7:116434729-116454408	19680	71	122	137	211
2	chr22:32920308-32927723	7416	53	72	94	109
3	chr7:115890993-115892266	1274	50	82	124	243
4	chr7:89787744-89795672	7929	49	92	85	108
5	chr7:115861595-115870968	9374	40	77	78	284
6	chr22:32170492-32188129	17638	40	64	96	102
7	chr22:32053085-32061138	8054	37	64	80	115
8	chr22:33262063-33266567	4505	37	87	60	112
9	chr21:34750664-34761738	11075	37	86	67	147

Table S2: Overlap of candidate loci among regions for cell line GM12878.

	chr7 region0	chr7 region1	chr7 region2	chr5 region3	chr5 region4	chr7 region5	chr7 region6	chr7 region7	chr22 region8	chr21 region9
region0	289	216	233	0	0	0	204	0	0	0
region1	216	290	233	0	0	0	209	0	0	0
region2	233	233	304	0	0	0	228	0	0	0
region3	0	0	0	130	99	0	0	0	0	0
region4	0	0	0	99	114	0	0	0	0	0
region5	0	0	0	0	0	156	0	128	0	0
region6	204	209	228	0	0	0	325	0	0	0
region7	0	0	0	0	0	128	0	190	0	0
region8	0	0	0	0	0	0	0	0	158	0
region9	0	0	0	0	0	0	0	0	0	231

Table S3: Overlap of candidate loci among regions for cell line K562.

	chr22 region0	chr22 region1	chr22 region2	chr21 region3	chr7 region4	chr7 region5	chrX region6	chr22 region7	chr22 region8	chr11 region9
region0	151	91	94	0	0	0	0	76	99	0
region1	91	154	65	0	0	0	0	62	84	0
region2	94	65	146	0	0	0	0	88	86	0
region3	0	0	0	189	0	0	0	0	0	0
region4	0	0	0	0	275	0	0	0	0	0
region5	0	0	0	0	0	153	0	0	0	0
region6	0	0	0	0	0	0	80	0	0	0
region7	76	62	88	0	0	0	0	129	70	0
region8	99	84	86	0	0	0	0	70	144	0
region9	0	0	0	0	0	0	0	0	0	116

Table S4: Overlap of candidate loci among regions for cell line HeLa.

	chr7 region0	chr7 region1	chr22 region2	chr7 region3	chr7 region4	chr7 region5	chr22 region6	chr22 region7	chr22 region8	chr21 region9
region0	305	192	0	200	0	220	0	0	0	0
region1	192	282	0	202 0	214	0	0	0	0	
region2	0	0	162	0	0	0	79	74	90	0
region3	200	202	0	293	0	238	0	0	0	0
region4	0	0	0	0	157	0	0	0	0	0
region5	220	214	0	238	0	324	0	0	0	0
region6	0	0	79	0	0	0	142	91	93	0
region7	0	0	74	0	0	0	91	152	94	0
region8	0	0	90	0	0	0	93	94	149	0
region9	0	0	0	0	0	0	0	0	0	184

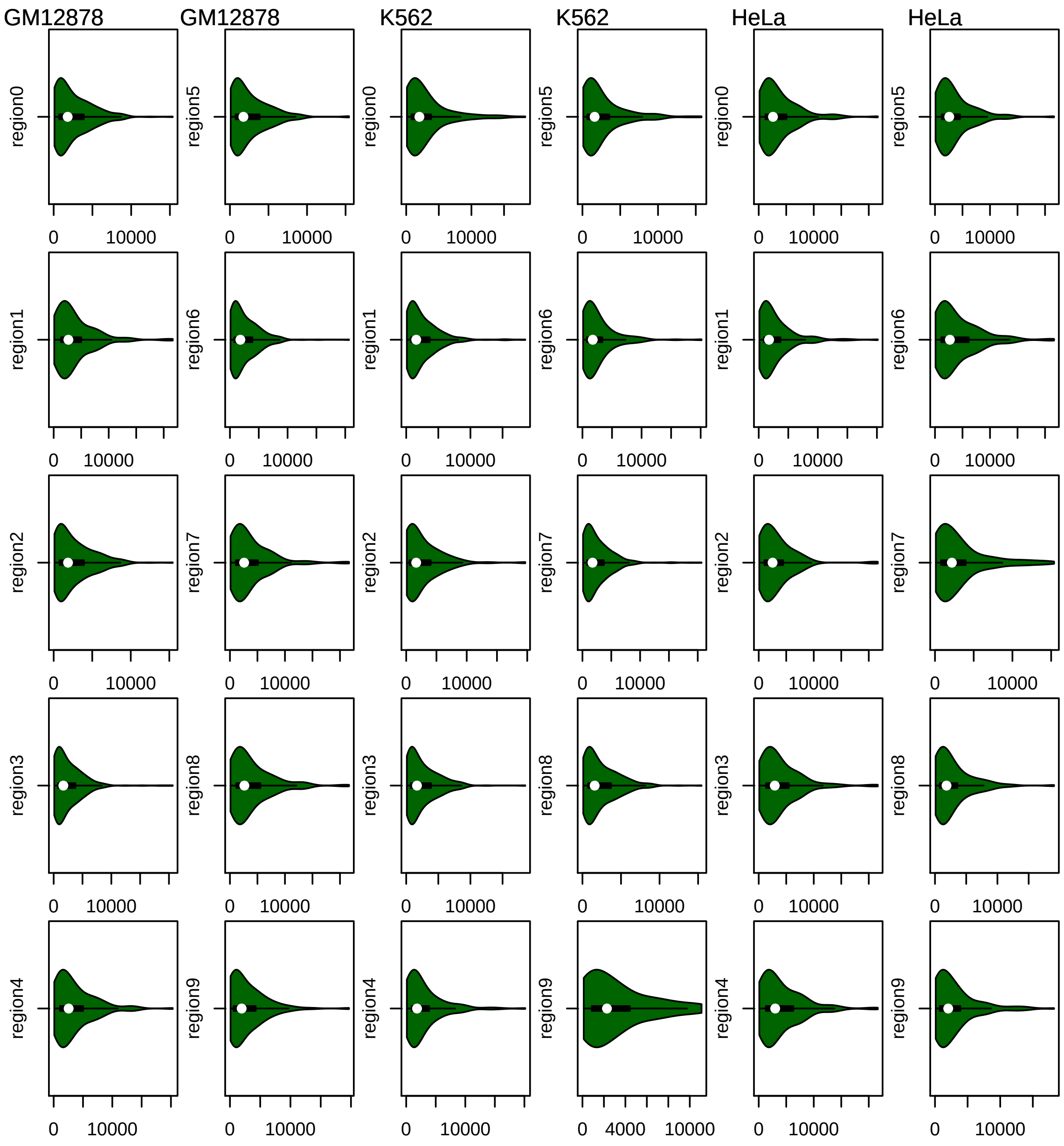

Figure S2: Lengths of restriction fragments for various *regions* in different cell lines. Their violin plots are arranged in two columns.

Performances

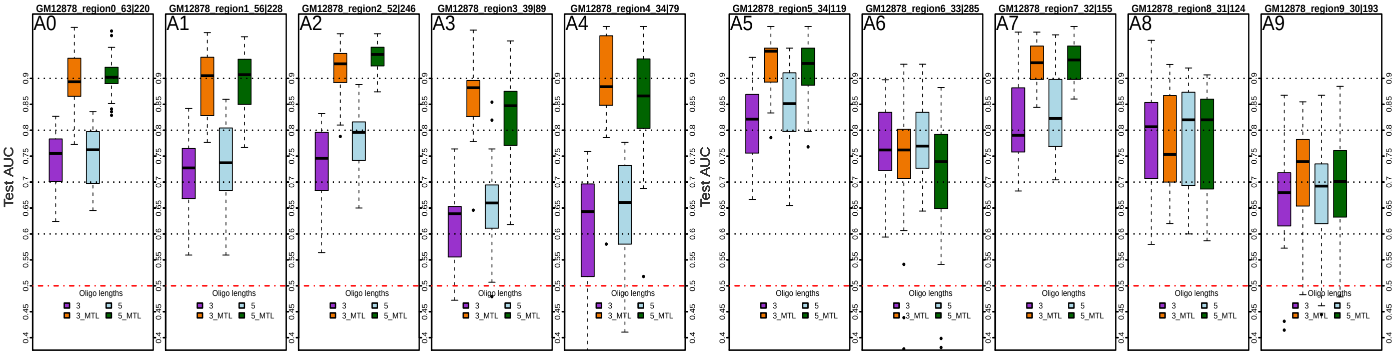

Figure S3: Box-plots of SVC performances for all *regions* (numbered ‘A0-A9’) in GM12878. Individual tasks setting, oligomer lengths = $\{3, 5\}$ in purple and light blue respectively. MTL with 10 tasks, oligomer lengths = $\{3, 5\}$ in orange and green. Distances between K-mer pairs upto $D = 100$.

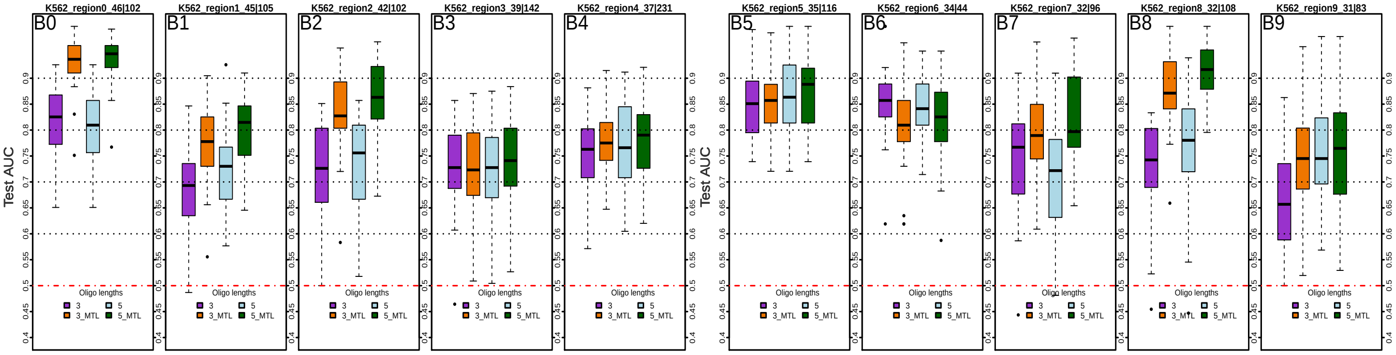

Figure S4: Box-plots of SVC performances for all *regions* (numbered ‘B0-B9’) in K562. Oligomer lengths = $\{3, 5\}$ in purple and light blue respectively. MTL with 10 tasks, oligomer lengths = $\{3, 5\}$ in orange and green. Distances between K-mer pairs upto $D = 100$.

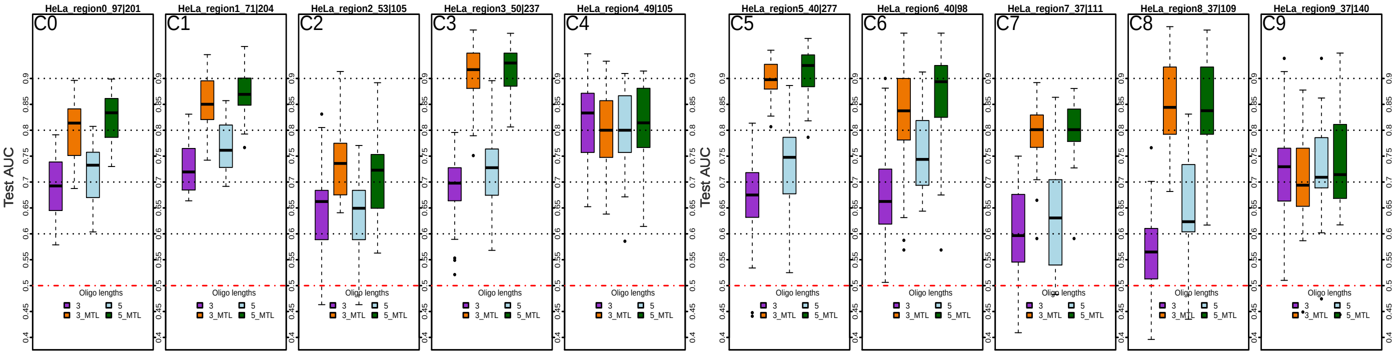

Figure S5: Box-plots of SVC performances for all *regions* (numbered ‘C0-C9’) in HeLa-S3. Oligomer lengths = $\{3, 5\}$ in purple and light blue respectively. MTL with 10 tasks, oligomer lengths = $\{3, 5\}$ in orange and green. Distances between K-mer pairs upto $D = 100$.

Visualizations

In the following we provide exemplar ‘AMPD’ and ‘Top25’ visualizations.

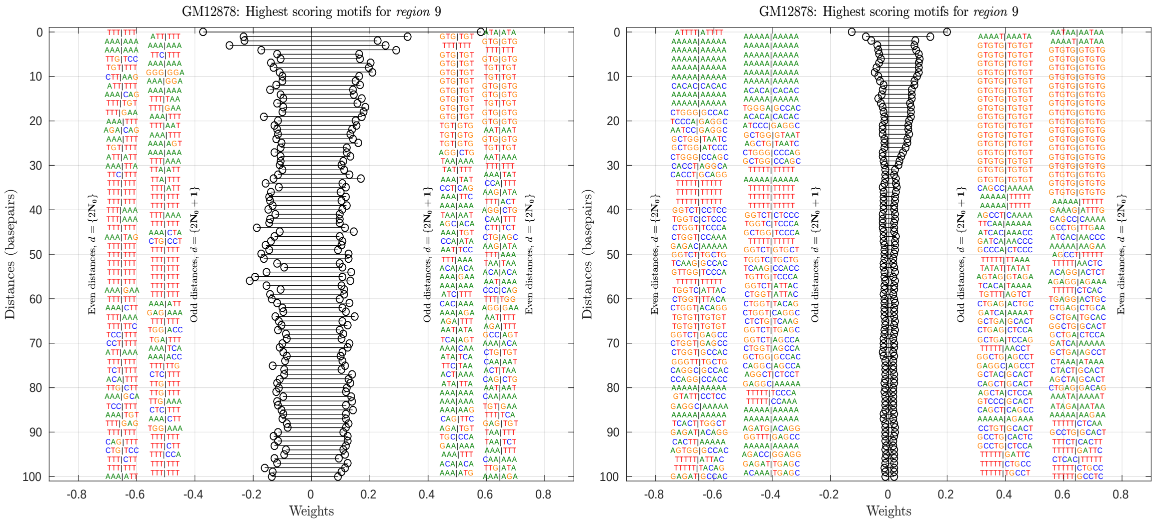

Figure S6: [Figure 3 in the main text reproduced here to ease comparison] ‘AMPD’ visualization of the informative K-mer pairs from the classifier for *region* 9 in GM12878 (Refer Table S1 for *region* details). Left panel: At distances in (0-100), the 3-mer pair that maximally contributes towards positive and negative classification of a given locus is shown. Weights are shown on the horizontal axis, distances on the vertical axis. Right panel: Visualization of the 5-mer case.

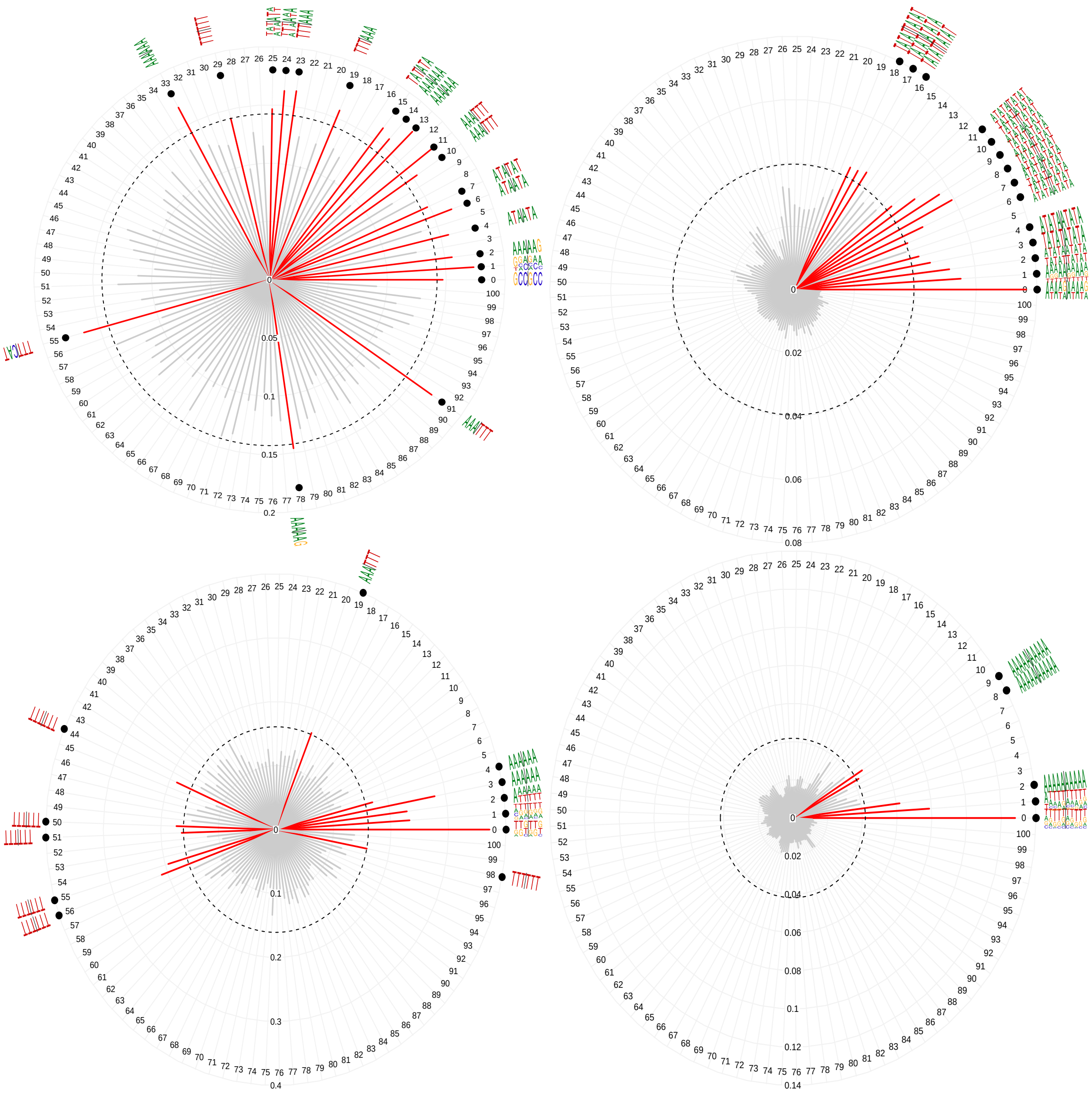

Figure S7: 'Top25' visualization of the informative 3-mer pairs separated by various distances and their magnitudes from the classifier for *region* 7 and 9 in GM12878 (Refer Table S1 for *region* details). Left: Top-25 3-mer pairs contributing to predicting a locus as belonging to the negative class (red); Right: Top-25 5-mer pairs. Dashed inner circle is the threshold to select the top-25 dimensions of the SVM weight vector.

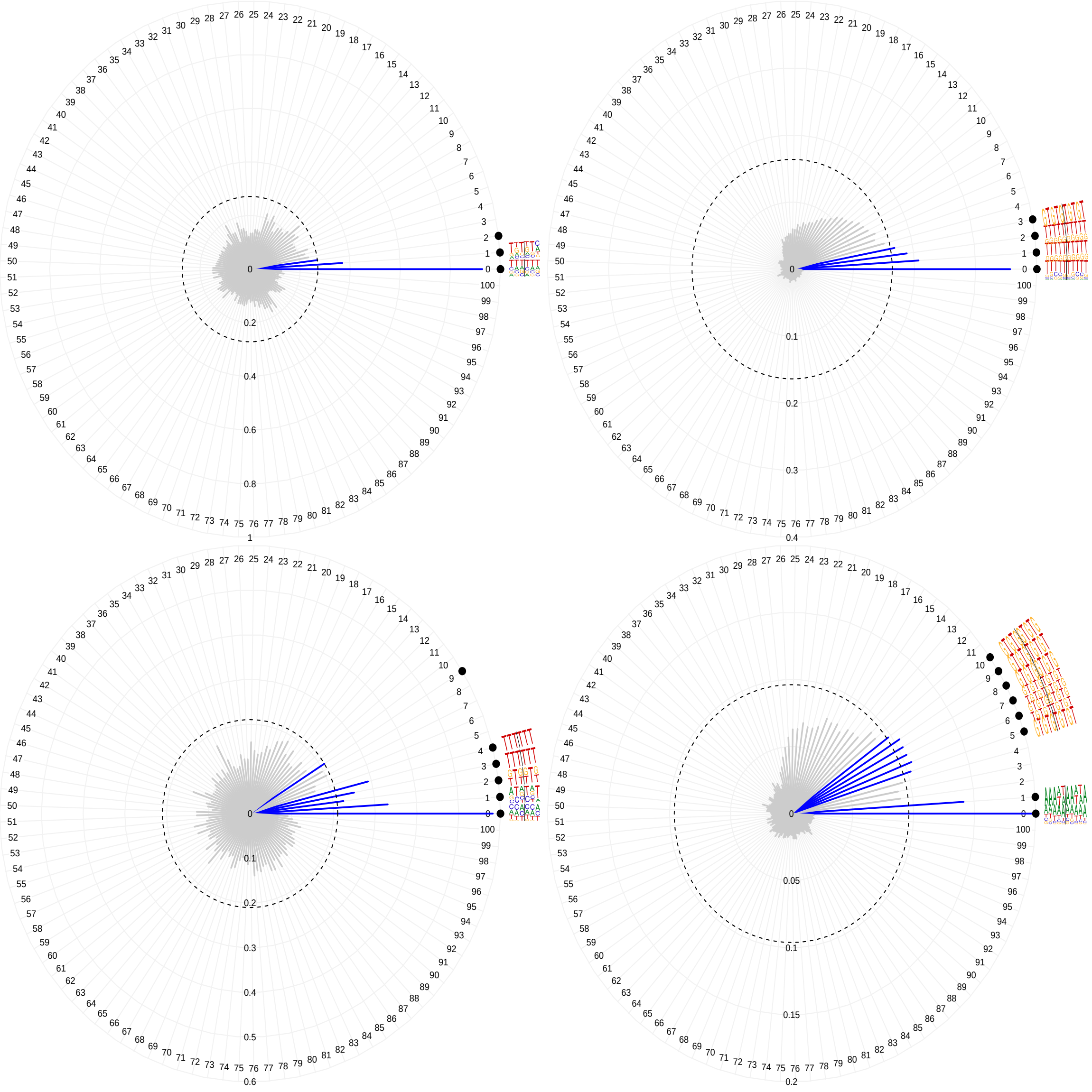

Figure S8: ‘Top25’ visualization of the informative 3-mer pairs separated by various distances and their magnitudes from the classifier for *region* 7 and 9 in GM12878 (Refer Table S1 for *region* details). Left: Top-25 3-mer pairs contributing to predicting a locus as belonging to the positive class (blue); Right: Top-25 5-mer pairs. Dashed inner circle is the threshold to select the top-25 dimensions of the SVM weight vector.

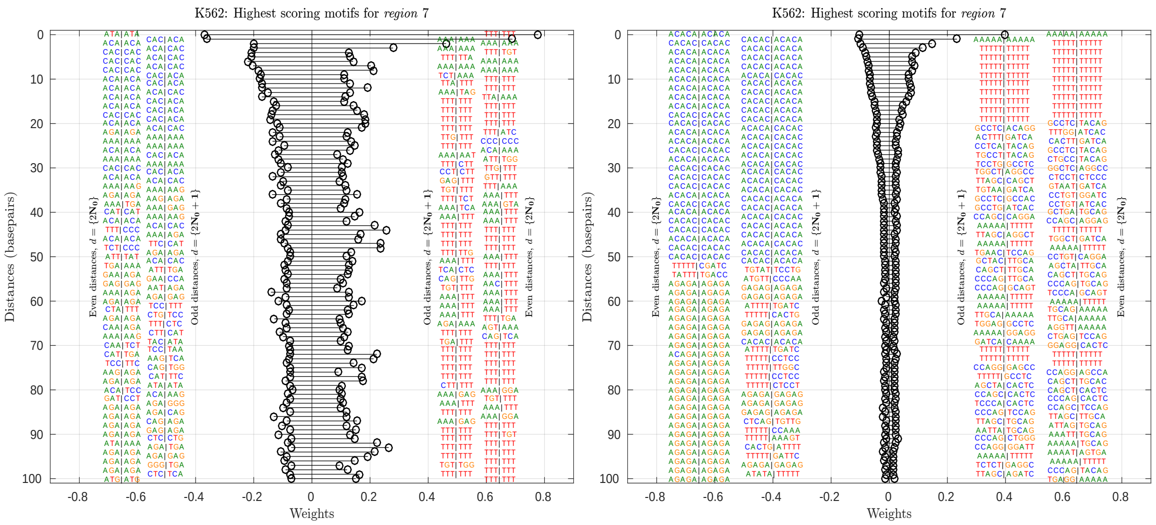

Figure S9: 'AMPD' visualization of the informative K-mer pairs from the classifier for *region* 7 in K562 (Refer Table S1 for *region* details). Left panel: At distances in (0-100), the 3-mer pair that maximally contributes towards positive and negative classification of a given locus is shown. Weights are shown on the horizontal axis, distances on the vertical axis. Right panel: Visualization of the 5-mer case. [The 3-mer case is reproduced from Figure 5 in the main text]

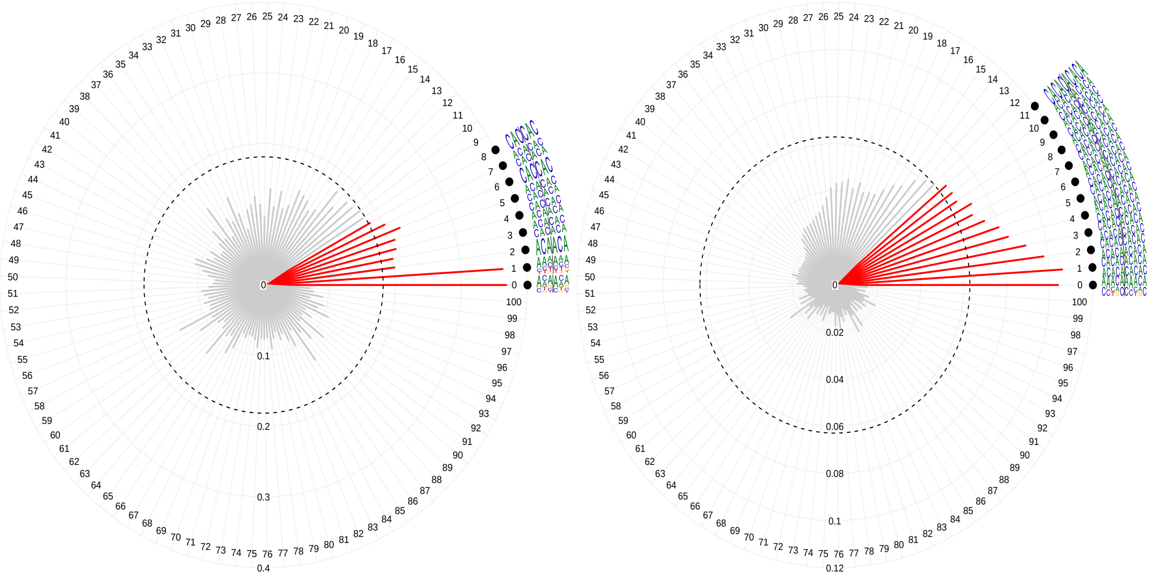

Figure S10: 'Top25' visualization of the informative 3-mer pairs separated by various distances and their magnitudes from the classifier for *region* 7 in K562 (Refer Table S1 for *region* details). Left: Top-25 3-mer pairs contributing to predicting a locus as belonging to the negative class (red); Right: Top-25 5-mer pairs. Dashed inner circle is the threshold to select the top-25 dimensions of the SVM weight vector.

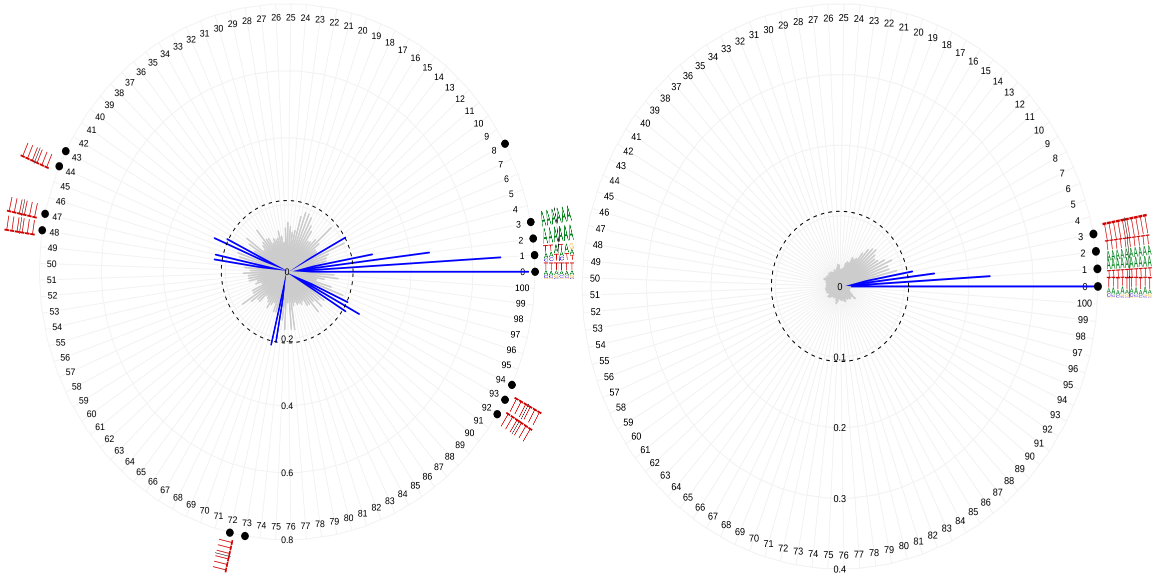

Figure S11: 'Top25' visualization of the informative 3-mer pairs separated by various distances and their magnitudes from the classifier for *region* 7 in K562 (Refer Table S1 for *region* details). Left: Top-25 3-mer pairs contributing to predicting a locus as belonging to the positive class (blue); Right: Top-25 5-mer pairs. Dashed inner circle is the threshold to select the top-25 dimensions of the SVM weight vector.

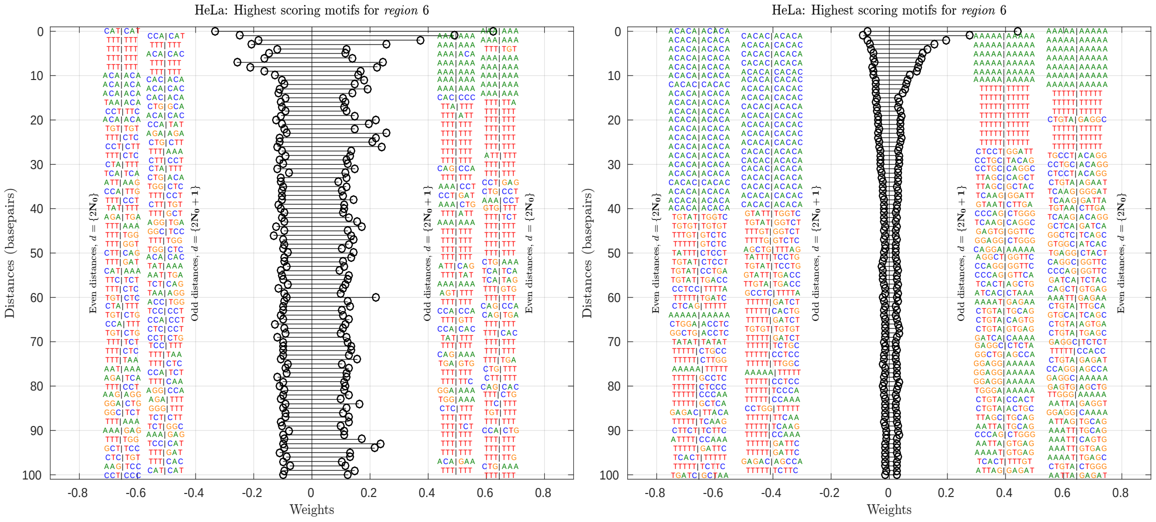

Figure S12: 'AMPD' visualization of the informative K-mer pairs from the classifier for *region* 6 in HeLa (Refer Table S1 for *region* details). Left panel: At distances in (0-100), the 3-mer pair that maximally contributes towards positive and negative classification of a given locus is shown. Weights are shown on the horizontal axis, distances on the vertical axis. Right panel: Visualization of the 5-mer case. [The 3-mer case is reproduced from Figure 5 in the main text]

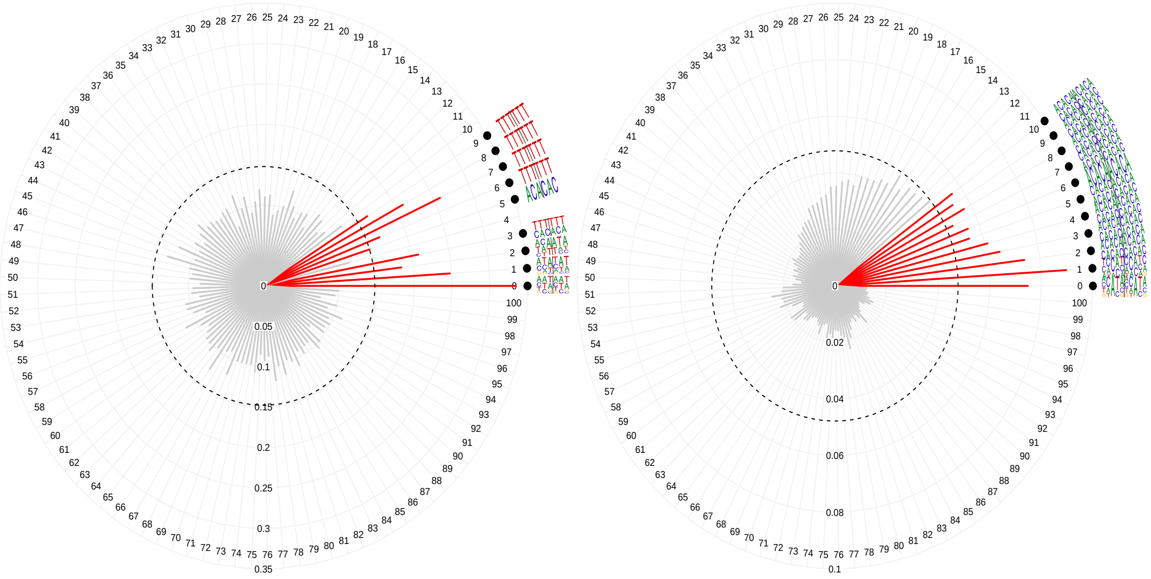

Figure S13: ‘Top25’ visualization of the informative 3-mer pairs separated by various distances and their magnitudes from the classifier for *region* 6 in HeLa (Refer Table S1 for *region* details). Left: Top-25 3-mer pairs contributing to predicting a locus as belonging to the negative class (red); Right: Top-25 5-mer pairs. Dashed inner circle is the threshold to select the top-25 dimensions of the SVM weight vector.

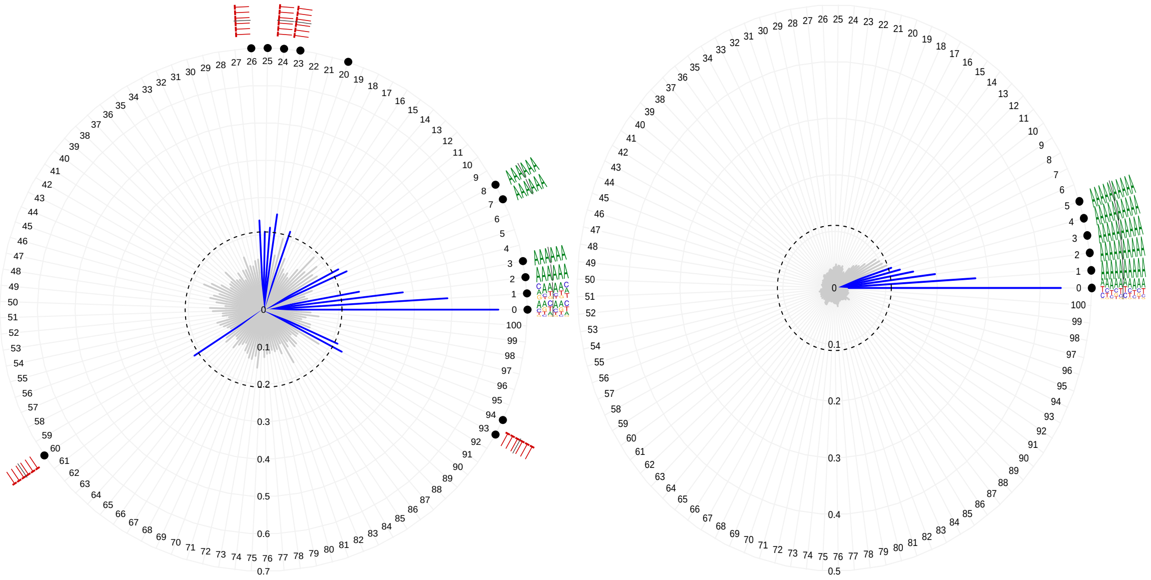

Figure S14: ‘Top25’ visualization of the informative 3-mer pairs separated by various distances and their magnitudes from the classifier for *region* 6 in HeLa (Refer Table S1 for *region* details). Left: Top-25 3-mer pairs contributing to predicting a locus as belonging to the positive class (blue); Right: Top-25 5-mer pairs. Dashed inner circle is the threshold to select the top-25 dimensions of the SVM weight vector.

References

[1] Amartya Sanyal et al. The long-range interaction landscape of gene promoters. *Nature*, 489(7414):109–113, Sep 2012.

[2] Jennifer Harrow et al. Gencode: The reference human genome annotation for the encode project. *Genome Research*, 22(9):1760–1774, 2012.

[3] Thomas Lingner and Peter Meinicke. Remote homology detection based on oligomer distances. *Bioinformatics (Oxford, England)*, 22(18):2224–2231, September 2006.

[4] Chih-Chung Chang and Chih-Jen Lin. LIBSVM: A library for support vector machines. *ACM Transactions on Intelligent Systems and Technology*, 2:27:1–27:27, 2011. Software available at `http://www.csie.ntu.edu.tw/%7Ecjlin/libsvm`.
